# Regulatory T Cells Function in Established Systemic Inflammation and Reverse Fatal Autoimmunity

**DOI:** 10.1038/s41590-021-01001-4

**Published:** 2021-08-23

**Authors:** Wei Hu, Zhong-Min Wang, Yongqiang Feng, Michail Schizas, Beatrice E. Hoyos, Joris van der Veeken, Jacob G. Verter, Regina Bou-Puerto, Alexander Y. Rudensky

**Affiliations:** 1 Howard Hughes Medical Institute, Immunology Program, and Ludwig Center, Memorial Sloan Kettering Cancer Center, New York, NY 10065, USA; 2 Gerstner Sloan Kettering Graduate School of Biomedical Sciences, Memorial Sloan Kettering Cancer Center, New York, NY 10065, USA; 3 Present address: Department of Immunology, St. Jude Children’s Research Hospital, Memphis, TN 38105, USA; 4 Present address: Research Institute of Molecular Pathology, Vienna BioCenter, Vienna, Austria; 5 Immunology and Microbial Pathogenesis Program, Weill Cornell Graduate School of Medical Sciences, New York, NY 10021, USA.

## Abstract

The immunosuppressive function of regulatory T (Treg) cells is dependent on continuous expression of the transcription factor Foxp3. *Foxp3* loss-of-function or induced ablation of Treg cells results in a fatal autoimmune disease featuring all known types of inflammatory responses with every manifestation stemming from Treg cell paucity, highlighting a vital function of Treg cells in preventing fatal autoimmune inflammation. However, a major question remains whether Treg cells can persist and effectively exert their function in a disease state, where a broad spectrum of inflammatory mediators can either inactivate Treg cells or render innate and adaptive pro-inflammatory effector cells insensitive to suppression. By reinstating Foxp3 protein expression and suppressor function in cells expressing a reversible *Foxp3* null allele in severely diseased mice, we found that the resulting single pool of “redeemed” Treg cells normalized immune activation, quelled severe tissue inflammation, reversed fatal autoimmune disease, and provided long-term protection against them. Thus, Treg cells are functional in settings of established broad spectrum systemic inflammation and are capable of affording sustained reset of immune homeostasis.

Regulatory T (Treg) cells expressing the X-linked transcription factor Foxp3 have been implicated in the control of inflammation in diverse settings^1–9^. Mice and humans lacking functional *Foxp3* gene develop fatal multi-organ autoimmune and inflammatory disease featuring lymphadenopathy and splenomegaly, eosinophilia, hyper IgE syndrome, and markedly increased systemic levels of a wide range of pro-inflammatory cytokines^1, 2, 3^. Foxp3 protein expression is required for Treg cell differentiation and function^4, 5, 6, 7^. Analyses of mice harboring a *Foxp3*^GFPKO^ reporter-null allele showed that Foxp3-deficient GFP^+^ Treg “wannabes” lack suppressor capacity^8, 9^, which can be restored by expression of a *Foxp3* transgene^10^. Foxp3-deficient mice display autoimmune pathology by day 10 of life with most mice dying within 3–4 weeks^5, 11, 12^. Likewise, Treg cell ablation induced upon diphtheria toxin (DT) treatment of healthy adult *Foxp3*^DTR-GFP^ mice, whose endogenous *Foxp3* locus encodes a simian DT receptor (DTR)-GFP fusion protein, leads to similar widespread autoimmune inflammation to which they succumb within 2 weeks^13^. Adoptive transfer of wild-type Treg cells into *Foxp3*^DTR-GFP^ mice at the time of DT administration or into 1-day-old *Foxp3* mutant mice prevents the disease^5, 13^. These studies have unequivocally demonstrated a critical role of Treg cells in preventing autoimmune inflammation and associated pathologies.

However, a major outstanding issue is whether Treg cells can effectively function in settings of established systemic inflammation and reverse and restrain severe autoimmune diseases. In fact, a large body of work in experimental animal models of infection, inflammation, and cancer suggests that major immune effector cytokines, including IL-6, IL-1, type I and II IFNs, IL-23, and IL-4, can cause Treg cells to lose Foxp3 expression, become functionally inactivated, and acquire pro-inflammatory features, or render immune effector cells refractory to Treg cell-mediated suppression^14, 15, 16, 17, 18, 19, 20^. Accordingly, numerous analyses of Treg cells present at inflammatory sites of autoimmune patients suggested their function was impaired^21, 22, 23, 24, 25^. Additional uncertainty stems from the possibility that the three archetypal inflammatory immune responses (types 1, 2, and 3), featuring distinct spectra of secreted and cellular effectors, may differ in their ability to compromise Treg cell functionality or sensitivity to Treg cell-mediated suppression. In this regard, variation in Treg cell frequencies was reported to result in a disproportionate dysregulation of type 2 vs. type 1 autoimmunity^26^. Thus, to address the major outstanding question above, we sought to employ an experimental animal model which would: 1) exhibit systemic mixed type 1, 2, and 3 autoimmunity and tissue inflammation; 2) enable temporal control and efficient switching of Treg cell suppressor function in an established disease; 3) eschew adoptive Treg cell transfer, which does not guarantee their sufficient migration to and accumulation at the inflammatory sites; and 4) allow for extended longitudinal observation.

We reason that the cardinal features of the aforementioned archetypal inflammatory responses are shared by a broad spectrum of autoimmune and inflammatory diseases. The breadth and severity of the autoimmune inflammation resulting from Foxp3 deficiency, characterized by activation and expansion of all major adaptive and innate immune cell types and rampant cytokine storm, make it an ideal experimental setting for testing the ability of Treg cells to operate in established inflammation. By engineering mice where an inducible Cre recombinase allowed for the installation of Treg cells in severely diseased animals, we found that Treg cells were functional in settings of established systemic mixed-type inflammation and capable of restoring health. Upon sensing the inflammatory environment, Treg cells became activated, rapidly expanded, exhibited heightened suppressive capacity, and persisted for an extended period of time with no signs of dysfunction.

## Results

### Restoration of Foxp3 expression in Treg “wannabes”

**Extended Data Figure 1 |:**
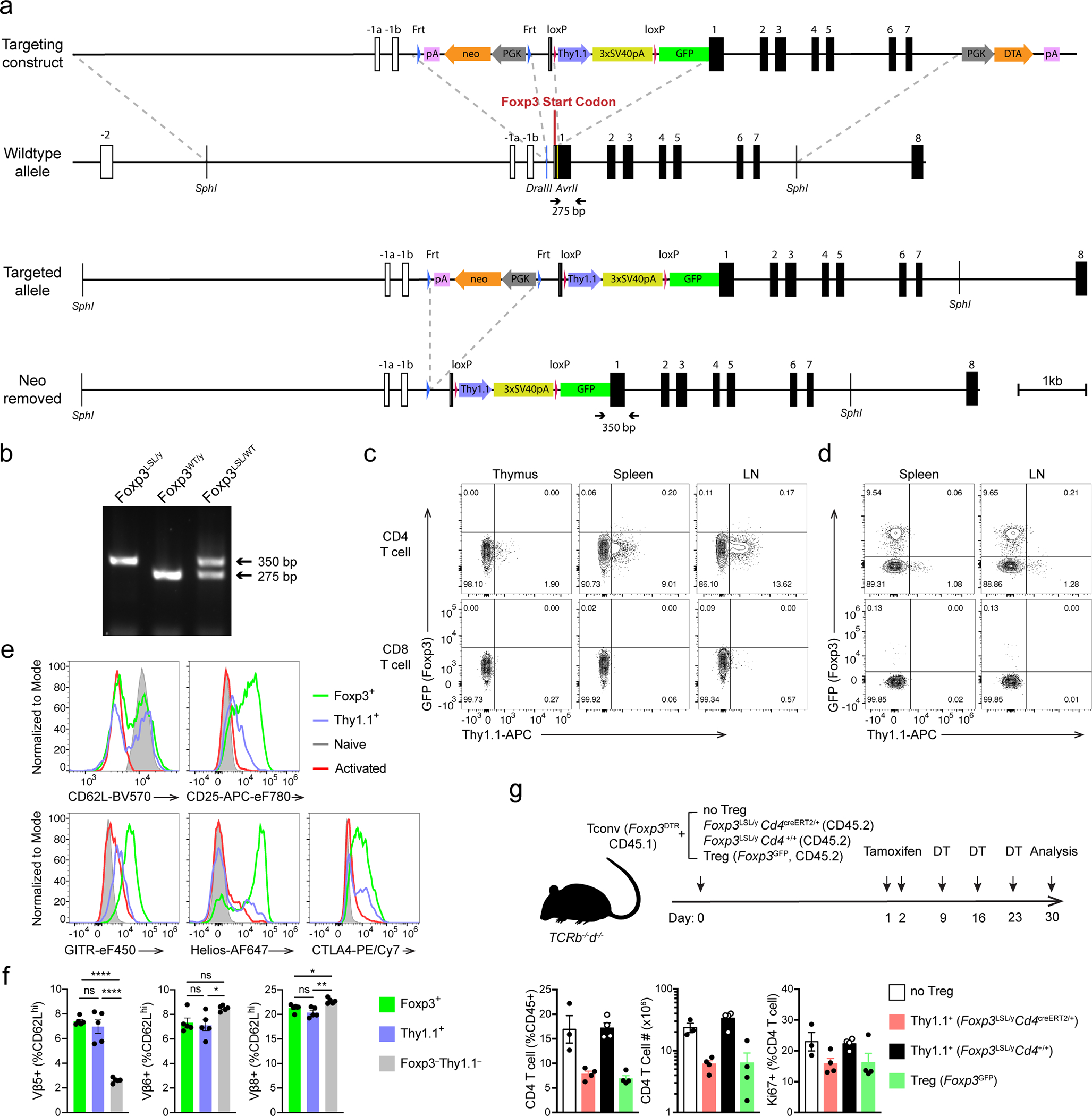
Generation and characterization of *Foxp3*^LSL^ mice. **a**, Schematics of the targeting construct and the *Foxp3*^LSL^ allele before and after Flp recombinase-mediated removal of the neo cassette. Homologous regions between the targeting construct and the WT allele are demarcated with dashed gray lines. Gene map is based on RefSeq record NM_001199347.1. pA, bovine growth hormone polyadenylation signal; neo, neomycin resistance gene; PGK, mouse phosphoglycerate kinase 1 promoter; 3xSV40pA, triple-tandem SV40 early polyadenylation signals (STOP cassette); DTA, diphtheria toxin A. **b**, Genotyping PCR showing the WT (275 bp) and knock-in-specific (350 bp) bands using primers labeled in **a**. **c, d**, Flow cytometric analysis of T cells and TCRβ^hi^ single positive thymocytes from 3-week-old male *Foxp3*^LSL/y^ (**c**) and 8–10-week-old female *Foxp3*^LSL*/*WT^ (**d**) mice. LN, lymph nodes. **e**, Expression of molecules associated with T cell activation in splenic Foxp3^+^ Treg and Thy1.1^+^ Treg “wannabes”, and naïve (CD44^lo^CD62L^hi^) and activated (CD44^hi^CD62L^lo^) conventional CD4 T cells shown in **d**. **f**, Percentages of TCRVβ5^+^, Vβ6^+^ and Vβ8^+^ cells among the indicated splenic CD4 T cell populations from 8–10-week-old female *Foxp3*^LSL*/*DTR-GFP^ mice. Cells are gated on the CD44^lo^CD62L^hi^ subset to avoid potential clonal expansion during activation. One way ANOVA. **g,** 2×10^6^ CD45.1^+^Foxp3^−^ conventional CD4 T cells were co-transferred with 2×10^5^ CD45.2^+^ Treg “wannabes” from 2–3-week-old *Foxp3*^LSL/y^ mice or Treg cells from 6–8-week-old *Foxp3*^*GFP*^ mice into *Tcrb*^*–/–*^*Tcrd*^*–/–*^ mice. Recipients were orally gavaged with tamoxifen and injected i.p. with diphtheria toxin (DT) at the indicated time points (top). DT was administered to deplete the few contaminating DTR-expressing Treg cells in the transferred FACS-purified conventional T cells or those induced to express Foxp3 after transfer. Cellularity and proliferation of CD45.1^+^ responder cells from lymph nodes were analyzed using flow cytometry (bottom). Data are representative of two independent experiments. All error bars denote mean ± s.e.m. ns, non-significant; *, p<0.05; **, p<0.01; ***, p<0.001; ****, p<0.0001.

**Figure 1 |:**
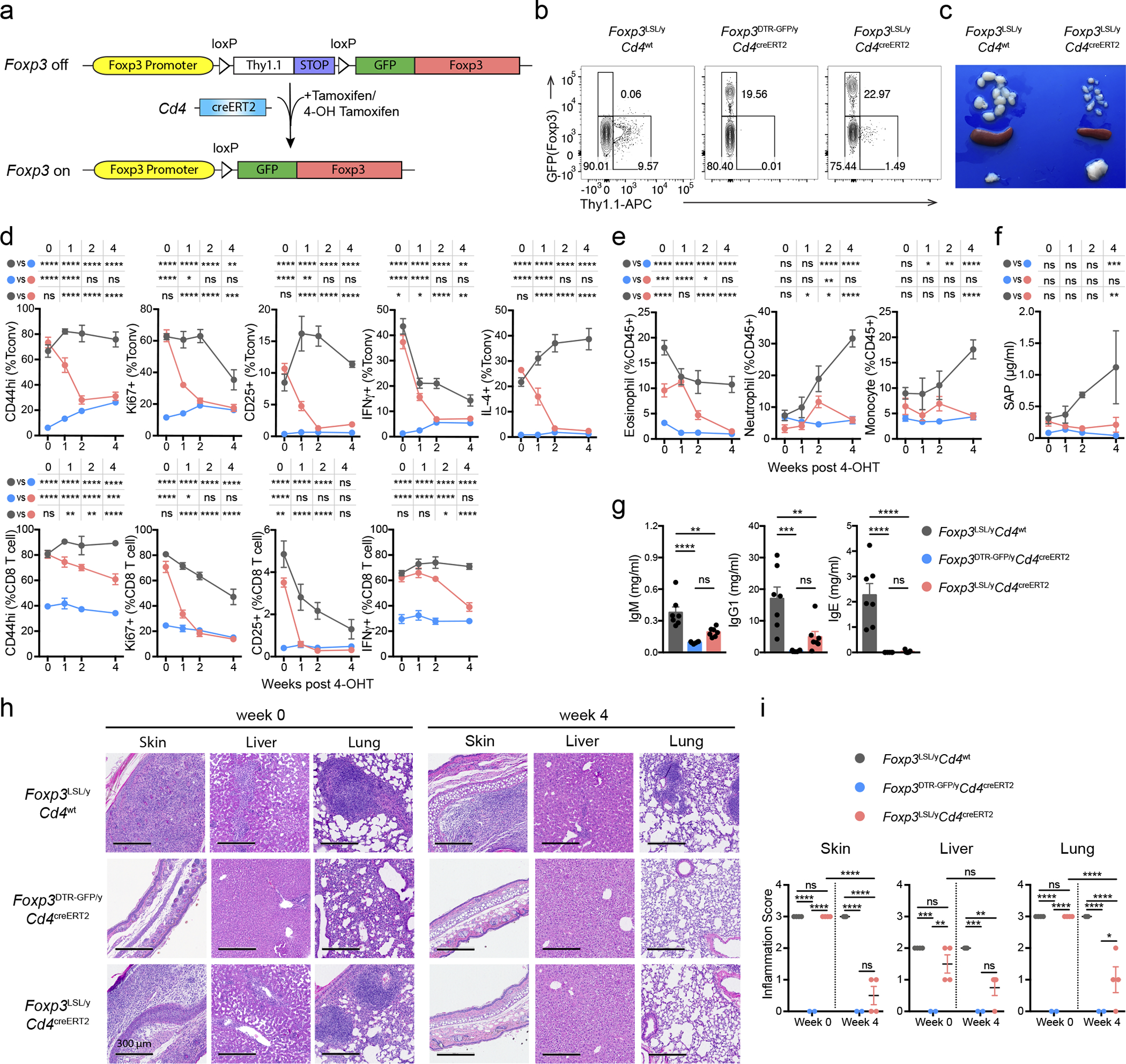
Restoration of *Foxp3* expression in Treg “wannabes” cures fulminant autoimmunity in male *Foxp3*^LSL^ mice. Mice were treated with 4-hydroxytamoxifen (4-OHT) on postnatal day 14. **a**, Schematic of the *Foxp3*^LSL^ allele. **b**, Flow cytometric analysis of splenic CD4 T cells 1 week post 4-OHT treatment. **c**, Lymph nodes (top), spleens (middle), and thymi (bottom) of mice of indicated genotypes 4 weeks after 4-OHT treatment. **d-f**, Percentages of activated, proliferating, and cytokine-producing splenic T cells (**d**), frequencies of splenic myeloid cell populations (**e**), and serum amyloid P (SAP) levels (**f**) at indicated time points after 4-OHT treatment. **g**, Levels of antibodies in the serum 4 weeks post-4-OHT treatment. **h,** Haematoxylin and eosin staining of indicated tissues before and 4 weeks after 4-OHT treatment. **i**, Histology scores of indicated tissues before and 4 weeks after 4-OHT treatment. For **d**-**g**, data are combined from three independent experiments with 3 to 12 mice per group per time point. Two-way (**d-f, i**) or one-way (**g**) ANOVA with Tukey’s multiple comparison test. All error bars denote mean ± s.e.m. ns, non-significant; *, p<0.05; **, p<0.01; ***, p<0.001; ****, p<0.0001

To investigate the ability of Treg cells to function in settings of established severe inflammation, we generated mice harboring a reversible *Foxp3* ^loxP-Thy1.1-STOP-loxP-GFP^ reporter-null allele (*Foxp3*^LSL^). In these mice, a loxP site-flanked Thy1.1 reporter followed by a STOP cassette was inserted into the *Foxp3* locus upstream of the *Foxp3*^GFP^ reporter allele with Thy1.1 expression marking a population of Treg “wannabes” (Extended Data Fig. 1a–d). These cells were phenotypically similar to those expressing the *Foxp3*^GFPKO^ allele^8^, and distinct from either naïve or activated conventional CD4 T cells, or Foxp3-sufficient Treg cells (Extended Data Fig. 1e). To ascertain that Treg “wannabes” bare a similarly self-reactive TCR repertoire as Treg cells, we assessed their TCR Vβ5 usage. C57BL/6 mice express viral superantigens vSAG8 and vSAG9, whose recognition by Vβ5 utilizing TCRs^27^ leads to partial deletion of Vβ5^+^ conventional CD4 T cells and promotes the generation of Vβ5^+^ Treg cells^28, 29^. We observed that Treg and “wannabe” cells showed comparably elevated Vβ5 usage relative to conventional CD4 T cells. This result suggests Treg and “wannabe” cells were similarly “self-focused” and that lack of Foxp3 did not result in shifted self-reactivity of the TCR repertoire of Treg “wannabes” (Extended Data Fig. 1f). Combining *Foxp3*^LSL^ allele with *Cd4*^creERT2^ allele encoding a tamoxifen-activatable Cre-ERT2 fusion protein enabled excision of the STOP cassette upon 4-hydroxytamoxifen (4-OHT) treatment and conversion of Foxp3-deficient Thy1.1^+^ “wannabes” into Foxp3-sufficient GFP^+^ Treg cells (Fig. 1a and 1b). The latter, when co-transferred with Foxp3^−^ CD4 T cells into lymphopenic recipients, suppressed the wasting disease similar to those isolated from control *Foxp3*^GFP^ mice (Extended Data Fig. 1g). Thus, rescued expression of endogenous Foxp3 in Treg “wannabes” conferred suppressor function and fitness in accordance with previous findings.

### Treg cells reverse established disease in young and adult mice

**Extended Data Figure 2 |:**
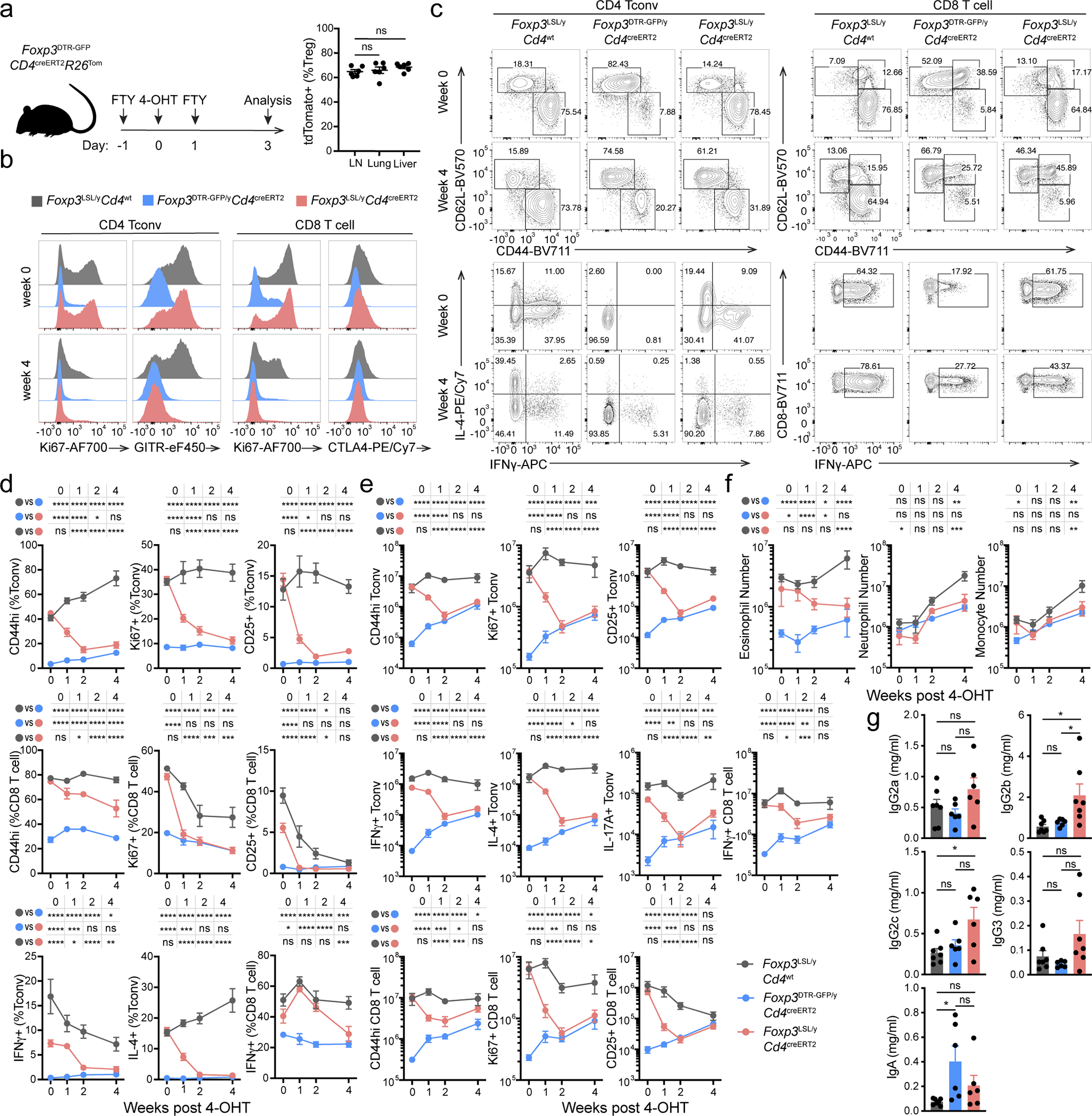
Restoration of *Foxp3* expression reverses spontaneous lymphocyte expansion, myelo-proliferation, and cytokine production, and normalizes circulatory Ig levels in male *Foxp3*^LSL^ mice. **a,** 4-OHT mediated recombination efficiency in lymphoid and non-lymphoid organs. Experimental scheme (left) and recombination efficiency of *Rosa26*^Tom^ in Treg cells from indicated organs (right). One-way ANOVA. LN, lymph nodes. **b-g,** Male *Foxp3*^LSL^ mice were treated with 4-OHT on postnatal day 14 and analyzed throughout the following 4 weeks. **b,** Representative histograms showing expression of proliferation and activation markers by splenic conventional CD4 and CD8 T cells at the indicated time points. **c,** Representative contour plots of flow cytometric analyses of activated and cytokine-producing splenic conventional CD4 and CD8 T cell populations. **d, e,** frequencies (**d**) and numbers (**e**) of activated, proliferating and cytokine-producing conventional CD4 and CD8 T cells from lymph nodes of mice of indicated genotypes at designated time points after 4-OHT treatment. Two-way ANOVA with Tukey’s multiple comparison correction. **f,** Numbers of splenic myeloid cell populations at indicated time points after 4-OHT treatment. Two-way ANOVA with Tukey’s multiple comparison correction. **g,** Serum antibody levels as in **Figure 1g.** One-way ANOVA with Tukey’s multiple comparison test. All error bars denote mean ± s.e.m. ns, non-significant; *, p<0.05; **, p<0.01; ***, p<0.001; ****, p<0.0001.

Hemizygous male *Foxp3*^LSL^ mice developed multi-organ autoimmune inflammation indistinguishable from that of Foxp3-deficient mice, to which they succumbed within 3–4 weeks with few surviving for up to 6 weeks after birth. To test whether Treg cells can suppress ongoing inflammation, male *Foxp3*^LSL^*Cd4*^creERT2^ or *Foxp3*^LSL^*Cd4*^wt^ mice were administered with a single dose of 4-OHT at 2 weeks of age, by which time they exhibited pronounced autoimmune syndrome with conspicuous clinical manifestations including blepharitis and macroscopic skin lesions. The recombination efficiency was comparable in lymphoid and non-lymphoid organs on day 3 following 4-OHT administration (Extended Data Fig. 2a). Within 4 weeks, the disease in 4-OHT-treated *Foxp3*^LSL^*Cd4*^creERT2^ mice was greatly alleviated with the extent of activation of innate and adaptive immune cells approaching the level of healthy *Foxp3*^DTR-GFP^*Cd4*^creERT2^ controls. In contrast, the disease in 4-OHT-treated *Foxp3*^LSL^*Cd4*^wt^ mice rapidly progressed with no mice surviving beyond 6 weeks of age (Fig. 1c–e; Extended Data Fig. 2b–f).

**Extended Data Figure 3 |:**
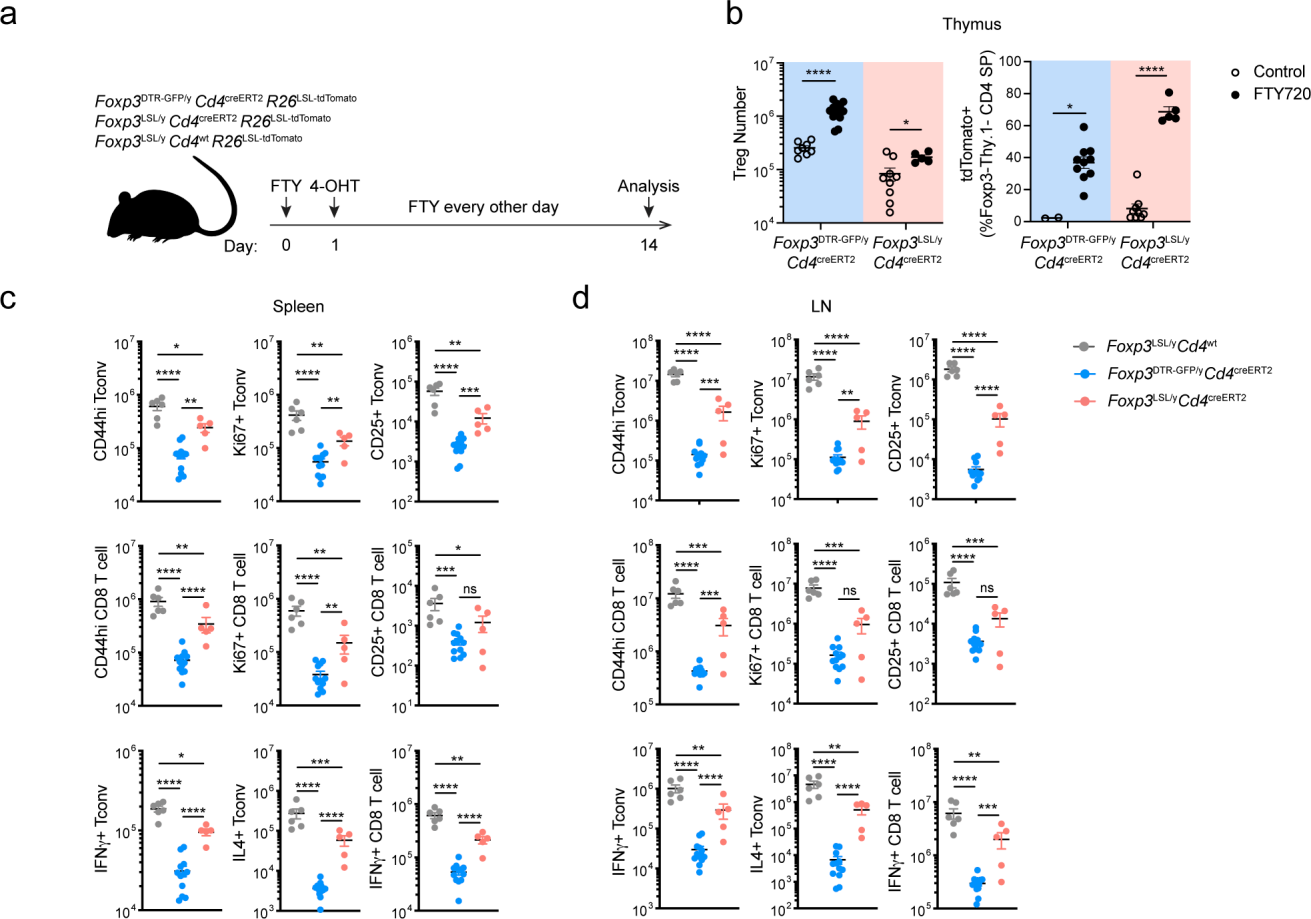
Restoration of *Foxp3* expression in peripheral Treg “wannabes” rescues immune activation in male *Foxp3*^LSL^ mice. **a,** Experimental Design. 2-week-old mice were treated with 4-hydroxytamoxifen (4-OHT) while being continuously treated with FTY720 to block thymic output. A *Rosa26*^*loxP-STOP-loxP-td*Tom*ato*^ (*R26*^LSL*-td*Tom*ato*^) recombination reporter allele was introduced to the mice to enable labeling of all CD4 T cells undergoing Cre-mediated recombination induced by 4-OHT. **b,** Number of Treg cells in the thymus (left), and percentage of tdTomato^+^ cells among Foxp3^−^Thy1.1^−^ CD4SP thymocytes (right), from FTY720-treated mice and untreated controls on day 14. Two-tailed unpaired *t*-tests with multiple hypothesis testing correction using the Holm-Sidak method. **c, d,** Numbers of activated, proliferating, and cytokine-producing conventional CD4 and CD8 T cells in the spleen (**c**) and lymph nodes (**d**). One-way ANOVA with Tukey’s multiple comparison test. All error bars denote mean ± s.e.m. ns, non-significant; *, p<0.05; **, p<0.01; ***, p<0.001; ****, p<0.0001.

**Extended Data Figure 4 |:**
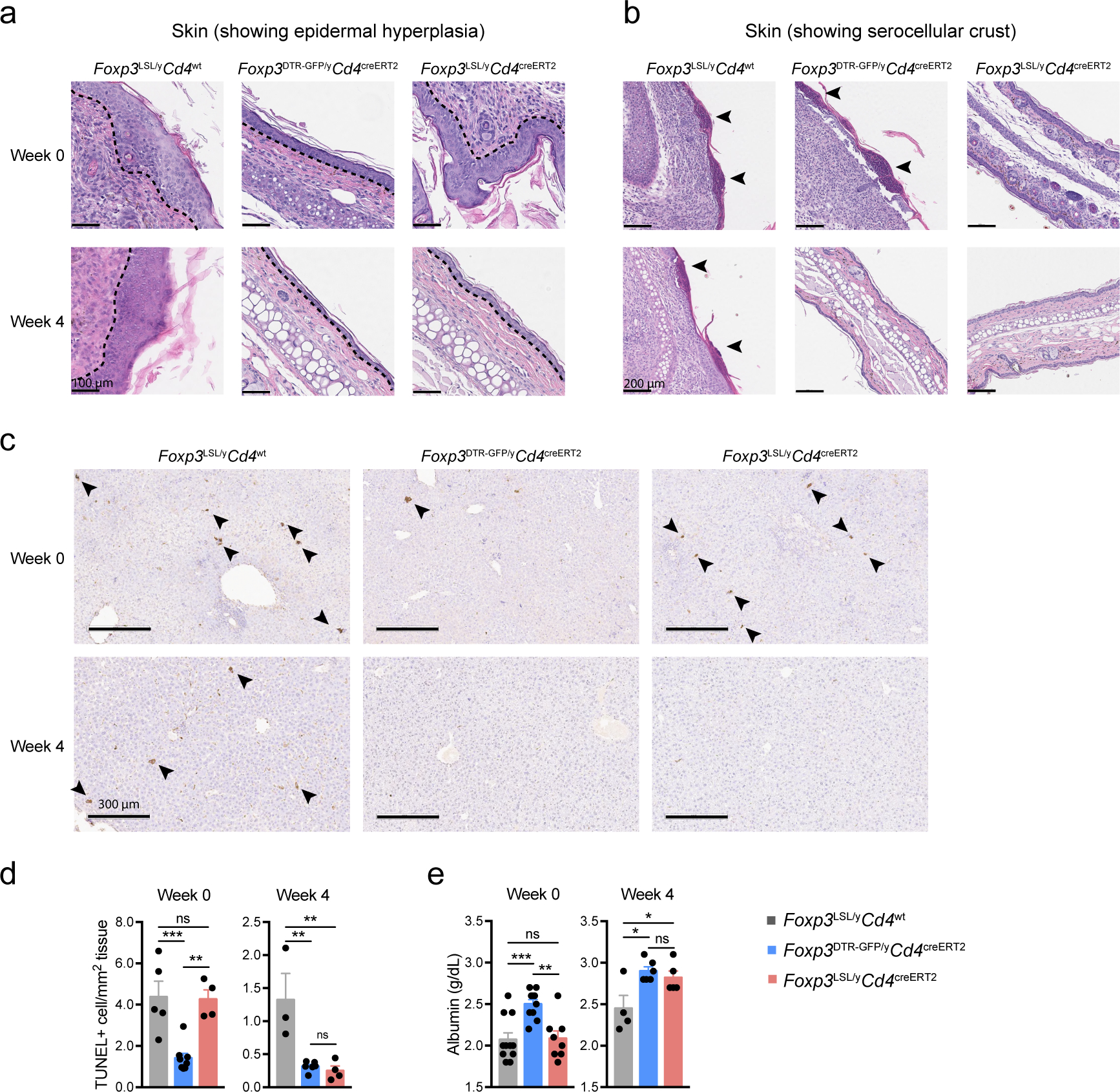
Restoration of *Foxp3* expression in Treg “wannabes” rescues tissue damage in the skin and liver of male *Foxp3*^LSL^ mice. Mice were treated with 4-OHT on postnatal day 14 and examined at the indicated time points post-treatment. **a, b,** H&E staining of skin sections showing epidermal hyperplasia and formation of serocellular crust (arrow heads). Dashed lines demarcate the boundary between epidermis and dermis. Images are representative of 4 *Foxp3*^LSL/y^*Cd4*^wt^, 4 *Foxp3*^LSL/y^*Cd4*^creERT2^ and 2 *Foxp3*^DTR-GFP/y^*Cd4*^creERT2^ mice. **c**, TUNEL (terminal deoxynucleotidyl transferase dUTP nick end labeling) assay followed by immunohistochemistry to visualize apoptotic cells (arrow heads) in H&E counter-stained liver sections from mice of indicated genotypes. **d,** Quantification of TUNEL^+^ cells. **e,** Measurement of serum albumin levels. All error bars denote mean ± s.e.m. ns, non-significant; *, p<0.05; **, p<0.01; ***, p<0.001; ****, p<0.0001.

Since the observed reversal of inflammation could be due to restoration of Foxp3 expression in thymocytes or peripheral Treg “wannabes”, we combined 4-OHT treatment with continuous FTY720-induced blockade of thymic output. Accordingly, we observed increased numbers of thymic Treg cells and the percentages of *Rosa26*^loxP-STOP-loxP-tdTomato^ (*R26*^Tom^) recombination reporter-expressing thymocytes labeled upon 4-OHT treatment (Extended Data Fig. 3a,b). Diminished immune activation and inflammation in *Foxp3*^LSL^*Cd4*^creERT2^ mice, compared to *Foxp3*^LSL^*Cd4*^wt^ controls, on day 14 following combined 4-OHT and FTY720 treatment suggested that Treg cells rescued in the periphery were effective at controlling inflammation in the absence of thymic output (Extended Data Fig. 3c–d). CD4 (Foxp3^−^Thy1.1^−^) and CD8 T cell activation was suppressed within a week after 4-OHT-induced restoration of Treg cell function, as evidenced by reduced expression of Ki67, CD44, CD25, IFNγ, and IL-4 (Fig. 1d; Extended Data Fig. 2d–e). Likewise, myelo-proliferation marked by expansion of neutrophils, monocytes, and eosinophils (Fig. 1e; Extended Data Fig. 2f) and severe acute phase response reflected in high level of serum amyloid P (SAP) were ameliorated in rescued *Foxp3*^LSL^*Cd4*^creERT2^ mice (Fig. 1f). In addition, levels of IgE, IgG1 and IgM were brought down by newly generated Treg cells in *Foxp3*^LSL^*Cd4*^creERT2^ mice in comparison to control *Foxp3*^LSL^*Cd4*^wt^ mice (Fig. 1g, Extended Data Fig. 2g). Manifest tissue immune infiltration and inflammation disappeared in *Foxp3*^LSL^*Cd4*^creERT2^ mice but not *Foxp3*^LSL^*Cd4*^wt^ controls within 4 weeks of 4-OHT administration (Fig. 1 h–i). A closer examination of the skin pathology in pre-treatment *Foxp3*^LSL^*Cd4*^creERT2^ and *Foxp3*^LSL^*Cd4*^wt^ mice revealed lichenoid interface dermatitis, which was resolved in treated *Foxp3*^LSL^*Cd4*^creERT2^ mice but continued to worsen in *Foxp3*^LSL^*Cd4*^wt^ mice (Extended Data Fig. 4a–b). Additionally, liver damage evident by apoptotic hepatocytes and decreased serum albumin concentrations in *Foxp3*^LSL^*Cd4*^creERT2^ mice normalized within 4 weeks after 4-OHT treatment (Extended Data Fig. 4c–e). The disease reversal was not a mere consequence of subtracting the Treg “wannabes” from the CD4 T cell pool, considering that the disease severity in Foxp3-deficient mice harboring Treg “wannabes” was identical to that of neonates subjected to chronic Treg cell ablation^13^. Thus, Treg cells are capable of effectively suppressing multiple arms of the inflammatory immune response in young mice under conditions of established autoimmunity, causing its reversal.

**Extended Data Figure 5 |:**
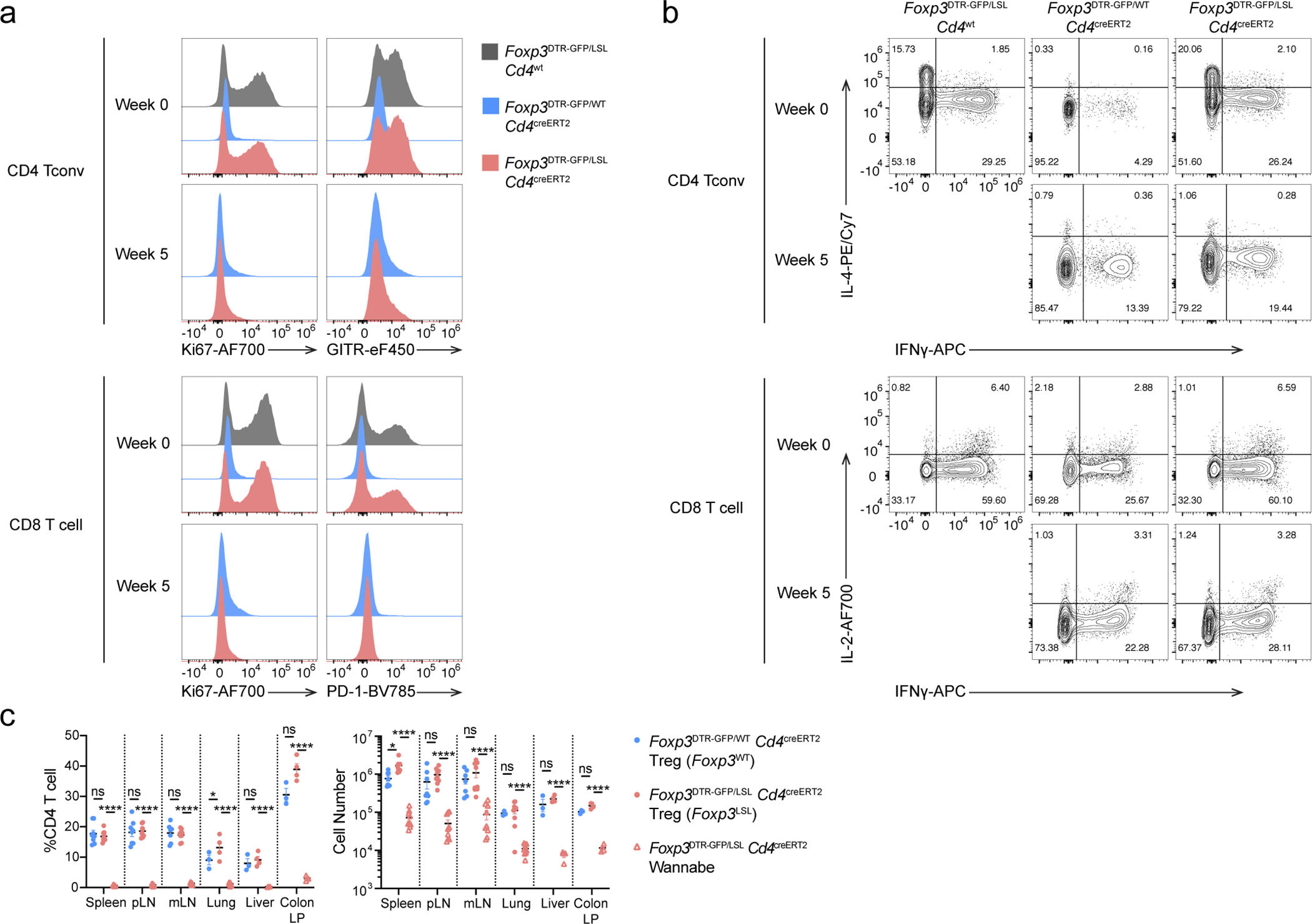
Restoration of *Foxp3* expression in Treg “wannabes” in mosaic adult female *Foxp3*^LSL*/*DTR-GFP^ mice suppresses immune activation caused by diphtheria toxin-mediated Treg cell ablation. Experimental scheme shown in Figure 2a. **a,** Representative histograms showing expression of activation and proliferation markers in splenic conventional T cell populations. **b,** Representative contour plots of splenic conventional CD4 and CD8 T cells showing cytokine production. **c,** Percentages (left) and numbers (right) of Treg and Treg “wannabes” from indicated tissues 5 weeks post 4-OHT administration. Two-way ANOVA with Tukey’s multiple comparison test. All error bars denote mean ± s.e.m. ns, non-significant; *, p<0.05; **, p<0.01; ***, p<0.001; ****, p<0.0001. pLN, peripheral (brachial, axillary, and inguinal) lymph nodes; mLN, mesenteric lymph nodes; LP, lamina propria.

**Figure 2 |:**
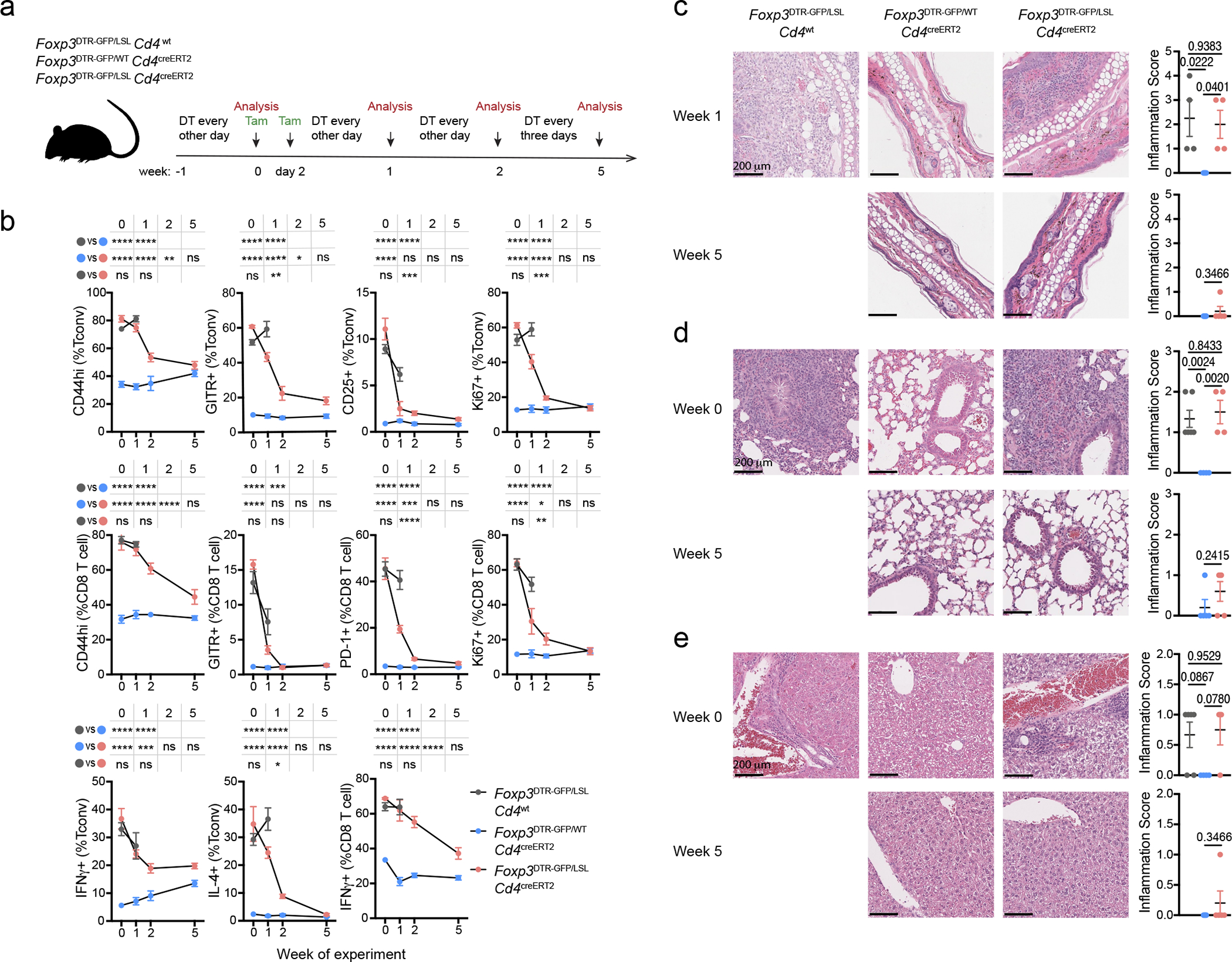
Restoration of *Foxp3* expression in Treg “wannabes” in mosaic adult female *Foxp3*^LSL*/*DTR-GFP^ mice suppresses immune activation caused by diphtheria toxin-mediated Treg cell ablation. **a,** Experimental scheme. 8–10-week-old female mice were injected with diphtheria toxin (DT) intraperitoneally and given oral gavage of tamoxifen (Tam) on designated days. **b,** Frequencies of activated, proliferating, and cytokine producing splenic conventional CD4 and CD8 T cells at the indicated time points. Data are pooled from two independent experiments with 3 to 5 mice per group per time point. Two-way ANOVA with Tukey’s multiple comparison test. **c-e,** Haematoxylin and eosin staining of sections of skin (**c**), lung (**d**), and liver (**e**) from mice of indicated genotypes at denoted time points post tamoxifen treatment (left), and their respective inflammation scores (right). One-way ANOVA with Tukey’s multiple comparison test. All error bars denote mean ± s.e.m. ns, non-significant; *, p<0.05; **, p<0.01; ***, p<0.001; ****, p<0.0001.

To assess whether Treg cells can restrain ongoing inflammation in adulthood, we generated healthy mosaic heterozygous female *Foxp3*^DTR-GFP*/*LSL^*Cd4*^creERT2^ mice, which harbor both a functional Treg and a non-functional “wannabe” population expressing the *Foxp3*^DTR-GFP^ and *Foxp3*^LSL^ allele, respectively. Recurrent DT administration causes widespread autoimmunity in *Foxp3*^DTR-GFP^ mice to which they succumb within 10–14 days^13^. Similarly, DT-treated adult (8–10-week-old) *Foxp3*^DTR-GFP*/*LSL^ heterozygous females exhibited massive immune activation by day 7, at which point the diseased *Foxp3*^DTR-GFP*/*LSL^*Cd4*^creERT2^ and *Foxp3*^DTR-GFP*/*LSL^*Cd4*^wt^ mice were administered tamoxifen to convert Treg “wannabes” to Treg cells (Fig. 2a). The rehabilitated Treg cells efficiently suppressed T cell activation within 2 weeks and ameliorated the inflammatory lesions in the skin, lung, and liver by week 5, whereas in control *Foxp3*^DTR-GFP*/*LSL^*Cd4*^wt^ mice the fatal disease rapidly progressed (Fig. 2b–e, Extended Data Fig. 5a–c). The 5 week endpoint was chosen as it was the longest period we were confident the mice were not developing neutralizing DT-specific antibodies. Thus, Treg cells can function under inflammatory settings and suppress established lethal autoimmunity in both neonates and adults.

### Foxp3 imparts Treg cell functionality in inflammatory milieu

**Figure 3 |:**
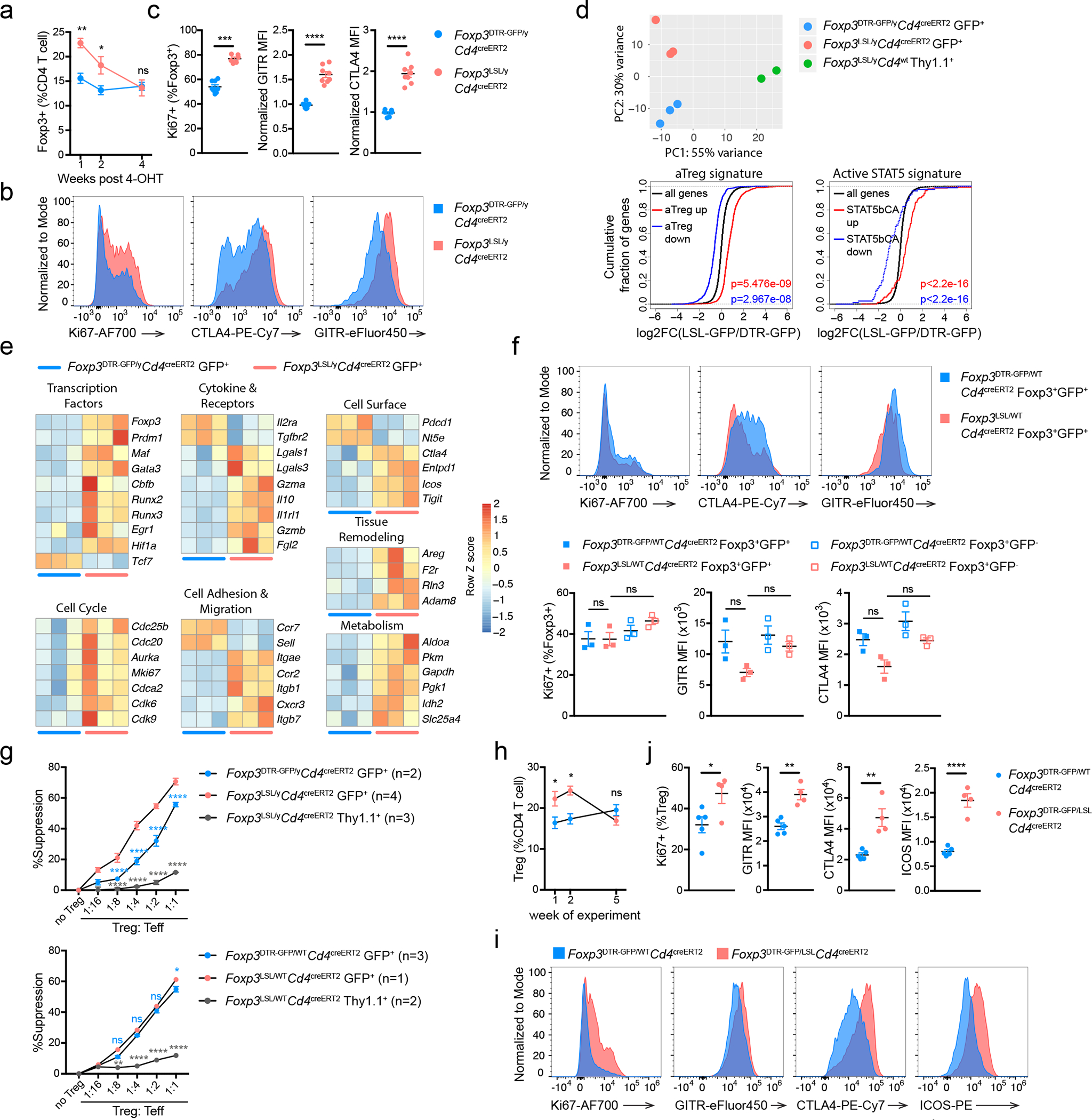
Rescued Treg cells in inflamed mice are activated and potently suppressive in inflammatory settings. **a-c**, Male *Foxp3*^DTR-GFP^*Cd4*^creERT2^ and *Foxp3*^LSL^*Cd4*^creERT2^ mice were treated with 4-OHT on postnatal day 14. Data are pooled from three independent experiments. **a**, Percentages of splenic Treg cells at indicated time points following 4-OHT treatment. Two-tailed unpaired *t*-tests with Holm-Sidak multiple comparison. **b, c**, representative histograms (**b**) and combined data (**c**) showing expression of Ki67, CTLA4 and GITR by rescued and control splenic Treg cells on day 7 post 4-OHT treatment. Two-tailed unpaired *t*-tests. **d, e**, RNA-seq analysis of rescued Treg cells, control Treg cells, and Treg “wannabes” from male mice of indicated genotypes treated with 4-OHT on postnatal day 14 and analyzed 7 days afterwards. **d**, Principal component analysis of gene expression in the three indicated cell populations (top), and empirical cumulative distribution function plots showing gene signatures of activated Treg cells (bottom left) and Treg cells expressing a constitutively active form of STAT5b (bottom right). Two-sided Kolmogorov-Smirnov test. **e**, Heatmaps showing expression of curated genes in rescued and control Treg cells from mice of indicated genotypes. **f**, Expression of Ki67, CTLA4 and GITR by indicated splenic Treg cell subsets from mosaic heterozygous *Foxp3*^DTR-GFP*/*WT^*Cd4*^creERT2^ and *Foxp3*^LSL*/*WT^*Cd4*^creERT2^ female mice treated with 4-OHT on postnatal day 14 and analyzed after 7 days. One-way ANOVA with Tukey’s multiple comparison test. **g**, Suppression of *in vitro* proliferation of conventional CD4 T cells induced by α-CD3 antibody and antigen-presenting cells by control or rescued Treg cells (GFP^+^) or Treg “wannabes” (Thy1.1^+^) from indicated strains of mice on day 7 post 4-OHT treatment. Two-way ANOVA with Tukey’s multiple comparison test. **h-j**, Adult mosaic heterozygous female *Foxp3*^DTR-GFP*/*WT^*Cd4*^creERT2^ and *Foxp3*^DTR-GFP*/*LSL^*Cd4*^creERT2^ mice were treated as in Figure 2a. **h**, Percentages of splenic Treg cells at indicated time points following tamoxifen treatment. Multiple *t*-tests with Holm-Sidak multiple comparison. **i, j,** Representative histograms (**i**) and combined data (**j**) showing expression of indicated markers by Treg cells at week 1. Two-tailed unpaired *t*-tests. All error bars denote mean ± s.e.m. ns, non-significant; *, p<0.05; **, p<0.01; ***, p<0.001; ****, p<0.0001.

Next, we explored the intrinsic and extrinsic phenotypic shifts Foxp3 expression afforded to Treg “wannabes” in inflammatory vs. non-inflammatory settings. Compared to healthy *Foxp3*^DTR-GFP^*Cd4*^creERT2^ littermates, we observed a transient increase in Treg proportion in male *Foxp3*^LSL^*Cd4*^creERT2^ mice, which peaked on day 7 post 4-OHT treatment and normalized after 4 weeks when the autoimmune disease was largely eradicated (Fig. 3a). On day 7, the Thy1.1^−^GFP^+^ Treg cells in rescued *Foxp3*^LSL^*Cd4*^creERT2^ mice were more proliferative and expressed higher amounts of GITR and CTLA4 than those in healthy *Foxp3*^DTR-GFP^*Cd4*^creERT2^ controls (Fig. 3b, c). RNA-seq analysis of Thy1.1^−^GFP^+^ Treg cells and Thy1.1^+^GFP^−^ “wannabes” isolated from *Foxp3*^LSL^ males on day 7 following 4-OHT treatment, and of Treg cells isolated from similarly treated *Foxp3*^DTR-GFP^*Cd4*^creERT2^ controls showed that Treg cells from diseased and control mice were considerably different (PC2; 30% variance), with differentially regulated genes in the former enriched for previously reported Treg cell^30^ and STAT5^31^ activation gene signatures (Fig. 3d). Notably, the transcriptomes of both Treg populations were markedly different—equidistant in PCA—from Thy1.1^+^GFP^−^ Treg “wannabes” (Fig. 3d). Treg cells in *Foxp3*^LSL^*Cd4*^creERT2^ mice downregulated *Tcf7*, *Sell*, and *Ccr7* transcripts and upregulated cell cycle, anabolism, and Treg suppressor function-related genes (*Gzma*, *Gzmb*, *Lgals1*, *Lgals3*, *Il10*, *Fgl2*, *Ctla4*, *Entpd1*, *Icos*, and *Tigit*)^32, 33^, suggesting that they were more metabolically active and suppressive than control Treg cells (Fig. 3e). Indeed, Treg cells isolated on day 7 post 4-OHT administration from *Foxp3*^LSL^*Cd4*^creERT2^ mice were significantly more potent at suppressing T cell proliferation *in vitro* than their counterparts from *Foxp3*^DTR-GFP^*Cd4*^creERT2^ mice (Fig. 3g).

**Extended Data Figure 6 |:**
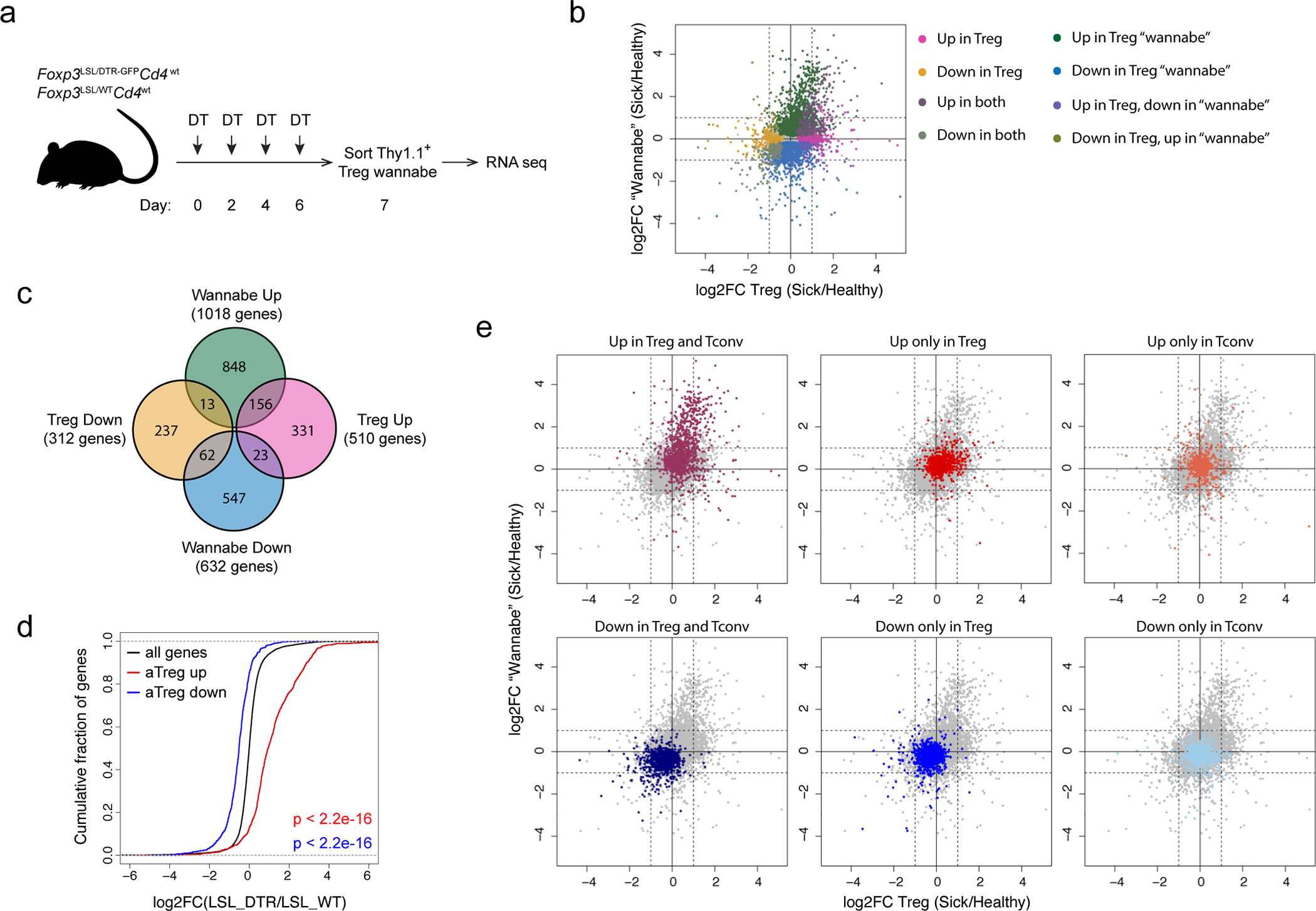
Analysis of gene expression changes in Treg “wannabes” induced upon activation. **a,** Experimental scheme. 8–10-week-old heterozygous female *Foxp3*^LSL*/*DTR-GFP^*Cd4*^wt^ mice were treated with diphtheria toxin (DT) on designated days to deplete *Foxp3*^DTR-GFP^-expressing Treg cells and induce activation of *Foxp3*^LSL^-expressing Treg “wannabes” which were sorted and analyzed by RNA-seq. **b,** FC-FC plot of activation-induced gene expression changes in Treg cells and “wannabes”. Genes with mean normalized counts of >100 are shown. Differentially expressed genes (*p*<0.05) are colored based on the direction of the change in either or both cell types. **c,** Venn diagram showing the numbers of genes with larger than a 2-fold change in activated Treg cells and “wannabes”. **d,** eCDF plots showing expression changes in activated Treg “wannabes” for all genes (black) and Treg activation signature genes that are up- (red) or down- (blue) regulated. Two-sided Kolmogorov-Smirnov test. **e,** FC-FC plots showing gene expression changes in Treg cells vs. “wannabes” isolated from sick and healthy mice. Signature genes that are up- or down-regulated in activated Treg and conventional CD4 T cells are highlighted in different colors.

Notably, activated Treg “wannabes” from DT-treated *Foxp3*^LSL*/*DTR-GFP^ sick female mice shared some gene expression changes with those of activated Treg cells in rescued *Foxp3*^LSL/y^ mice (Fig. 3d, Extended Data Fig. 6). However, this acquisition of activated phenotype by Treg “wannabes” did not confer any appreciable suppressive capacity, as *Foxp3*^LSL*/DTR*^*Cd4*^wt^ mice, in which tamoxifen treatment could not restore Foxp3 expression, died of similar systemic multi-organ inflammation seen in *Foxp3*^LSL/y^ mice. Thus, the heightened suppressor capacity of rehabilitated Treg cells in *Foxp3*^LSL^*Cd4*^creERT2^ mice could be due to exposure to an inflammatory environment upon *Foxp3* induction or a cell-intrinsic effect of delayed Foxp3 expression regardless of the environment. To distinguish between these two possibilities, we analyzed the “wannabe”-converted and control Treg cells under basal non-inflammatory conditions in healthy heterozygous female *Foxp3*^LSL*/*WT^*Cd4*^creERT2^ and *Foxp3*^DTR-GFP*/*WT^*Cd4*^creERT2^ mice, respectively, on day 7 post 4-OHT treatment. In contrast to Treg cells exposed to the inflammatory environment in male mice, Thy1.1^−^GFP^+^ Treg cells in 4-OHT-treated mosaic *Foxp3*^LSL*/*WT^*Cd4*^creERT2^ females failed to exhibit increased proliferative activity or enhanced CTLA4 and GITR expression, compared to GFP^−^Foxp3^+^ Treg cells expressing the *Foxp3*^WT^ allele in the same mice (Fig. 3f). Likewise, comparable suppressor activity was observed for Thy1.1^−^GFP^+^ Treg cells and control GFP^+^ counterparts isolated from 4-OHT-treated healthy *Foxp3*^LSL*/*WT^*Cd4*^creERT2^ and *Foxp3*^DTR-GFP*/*WT^*Cd4*^*cr*e*ERT2*^ female mice, respectively (Fig. 3g). To the contrary, sick *Foxp3*^DTR-GFP*/*LSL^*Cd4*^creERT2^ mosaic female mice, subjected to DT-induced ablation of *Foxp3*^DTR-GFP^ Treg cells and tamoxifen-induced Foxp3 restoration in *Foxp3*^LSL^ cells, showed a transient increase in Treg percentages at 1 and 2 weeks post tamoxifen-mediated rescue, which disappeared by week 5 (Fig. 3h). These Treg cells also exhibited enhanced proliferation and heightened activation as evidenced by elevated expression of Ki67, CTLA4, GITR and ICOS (Fig. 3i, j). Thus, the enhanced proliferative potential and suppressive capacity of Treg cells in diseased *Foxp3*^LSL^*Cd4*^creERT2^ male and DT-treated *Foxp3*^DTR-GFP*/*LSL^*Cd4*^creERT2^ female mice were likely due to sensing of inflammation assisted by restored Foxp3 expression.

### A single cohort of Treg cells provides long-term protection

**Extended Data Figure 7 |:**
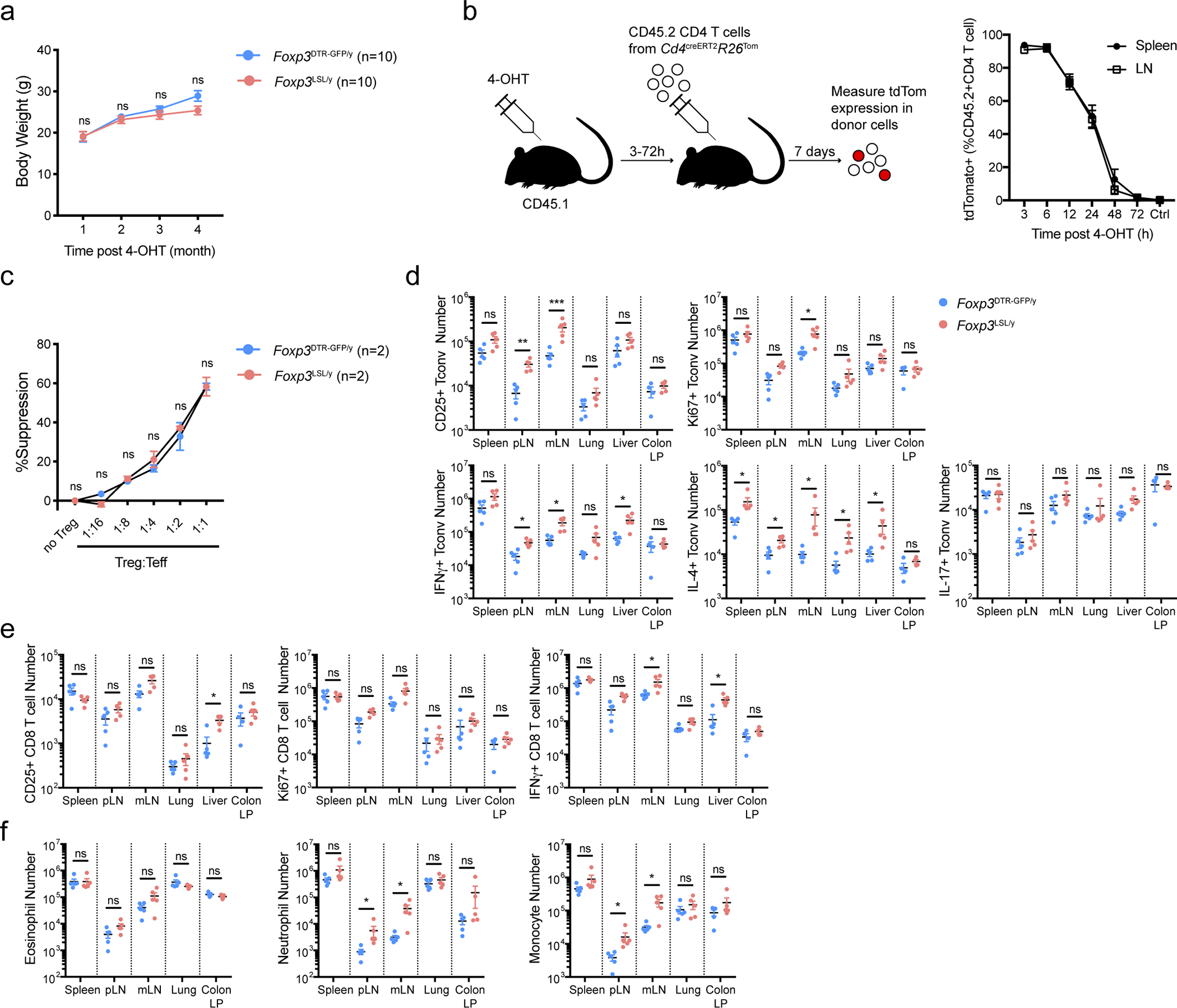
Rescued Treg cells in male *Foxp3*^LSL^ mice exert long-term control of adaptive and innate immune cells. Mice were treated with a single dose of 4-OHT at 2 weeks of age and analyzed 4 months later. **a,** Body weight of rescued *Foxp3*^LSL^*Cd4*^creERT2^ and control *Foxp3*^DTR-GFP^*Cd4*^creERT2^ mice over a 4-month time course. Two-way ANOVA with Sidak’s multiple comparison test. **b,** Analysis of 4-OHT “functional” pharmacokinetics in 2-week-old male mice. Mice were injected with 4-OHT 3–72 hours before transfer of congenically marked recombination-proficient CD4 T cells from *Cd4*^creERT2^*R26*^Tom^ mice. 4-OHT-induced recombination was assayed 7 days later by tdTomato expression among donor CD4 T cells as a readout for 4-OHT activity. Data are pooled from two independent experiments with 2 to 4 mice per time point each. Ctrl, no 4-OHT injection. **c,** Suppression of *in vitro* proliferation of conventional CD4 T cells induced by α-CD3 antibody and antigen-presenting cells by control Treg cells from *Foxp3*^DTR-GFP/y^ or rescued Treg cells from *Foxp3*^LSL/y^ mice. Two-way ANOVA with Tukey’s multiple comparison test. **d-f,** Numbers of activated, proliferating, and cytokine-producing conventional CD4 T cells (**d**), CD8 T cells (**e**) and myeloid populations (**f**) in indicated tissues. pLN, peripheral (brachial, axillary, and inguinal) lymph nodes; mLN, mesenteric lymph nodes; LP, lamina propria. Data are pooled from two independent experiments. Two-tailed *t*-tests with multiple hypothesis testing correction using the Holm-Sidak method. All error bars denote mean ± s.e.m. ns, non-significant; *, p<0.05; **, p<0.01; ***, p<0.001; ****, p<0.0001.

**Extended Data Figure 8 |:**
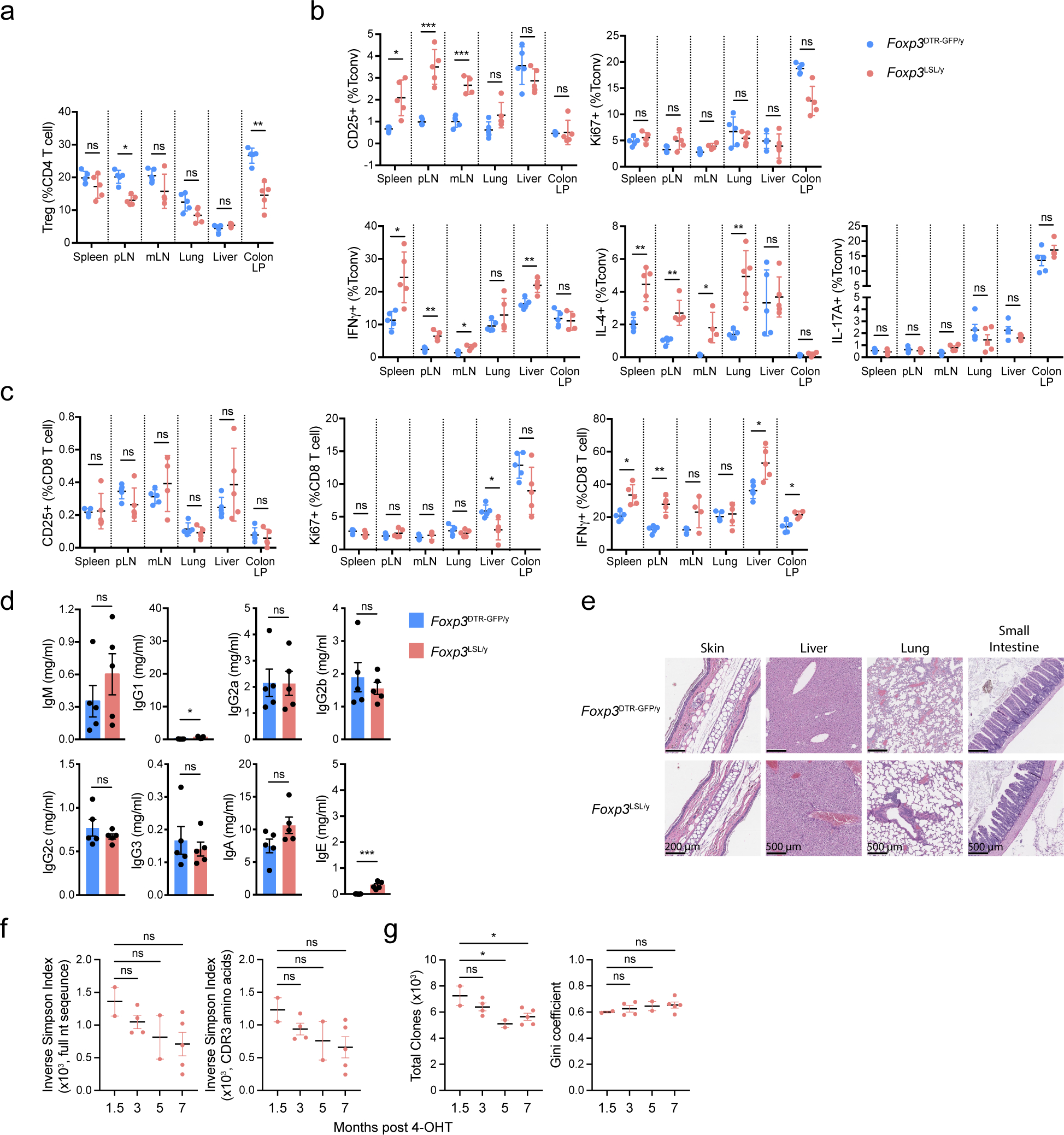
Rescued Treg cell population persisting for 7 months in male *Foxp3*^LSL^ mice prevents relapse of rampant autoimmunity. Mice were treated with 4-OHT on postnatal day 14 and analyzed 7 months later. **a-c,** Frequencies of Treg cells (**a**) and proliferating, activated, and cytokine-producing conventional CD4 (**b**) and CD8 (**c**) T cells. Two-tailed unpaired *t*-tests with multiple hypothesis testing correction using the Holm-Sidak method. pLN, peripheral (brachial, axillary, and inguinal) lymph nodes; mLN, mesenteric lymph nodes; LP, lamina propria. **d,** Serum antibody levels. Scales were kept the same as in Figure 1g. Two-tailed unpaired *t*-test. **e,** Representative images of haematoxylin and eosin-stained sections of the indicated organs. Images are representative of 5 *Foxp3*^DTR-GFP/y^ and 5 *Foxp3*^LSL/y^ mice. **f**, Clonal diversity of the TCRα repertoire of the long-lived “redeemed” Treg cells from *Foxp3*^LSL/y^ *Cd4*^creERT2^ mice at indicated time points after restoring Foxp3 expression upon 4-OHT treatment. The inverse Simpson Index was calculated based on the clone size distribution using clonotypes defined by full nucleotide sequence (left) or CDR3 amino acid sequence (right). **g**, Total number of unique clones (left) and Gini coefficient (right) of the TCRα repertoire of the long-lived “redeemed” Treg cells. Clonotypes were defined by using the full nucleotide sequence. One-way ANOVA with Dunnett’s multiple hypothesis test. All error bars denote mean ± s.e.m. ns, non-significant; *, p<0.05; **, p<0.01; ***, p<0.001; ****, p<0.0001.

**Figure 4 |:**
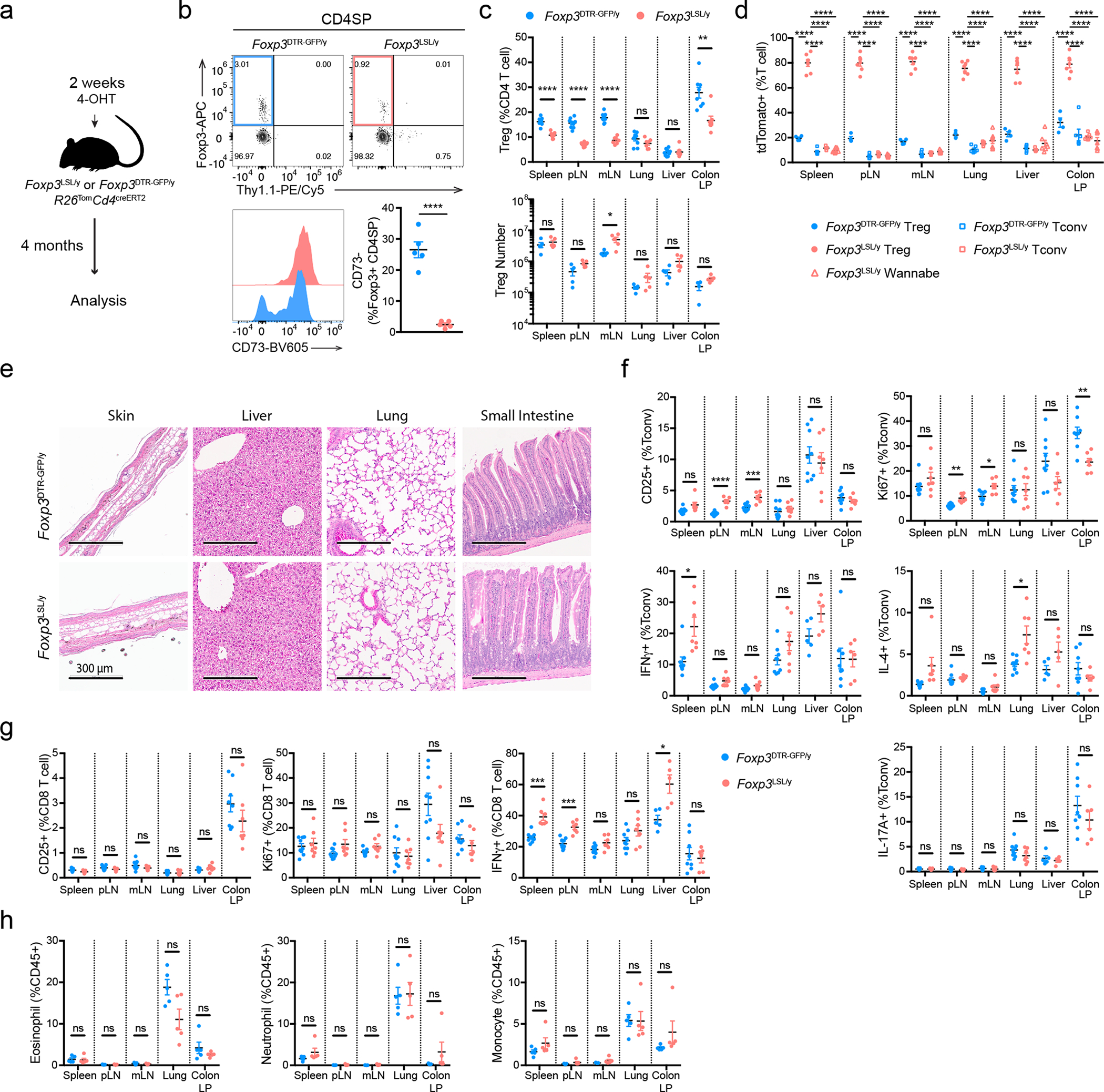
Rescued Treg cells in male *Foxp3*^LSL^ mice provide long-term protection from autoimmune inflammatory disease. **a,** Experimental design. Mice were treated with a single dose of 4-OHT at 2 weeks of age and analyzed 4 months later. **b,** Flow cytometric analysis of CD73 expression in Foxp3^+^ CD4 single-positive thymocytes as a discriminating marker of recirculating vs. recently generated thymic Treg cells. Two-tailed unpaired *t*-test. **c,** Flow cytometric analysis of Treg cell percentages (upper panel) and absolute numbers (lower panel) in tissues of mice of indicated genotypes. Two-tailed unpaired *t*-tests with Holm-Sidak multiple comparison. **d,** Percentages of lineage-traced (tdTomato^+^) Treg cells, Treg “wannabes”, and conventional CD4 T cells. One-way ANOVA with Dunnett’s multiple hypothesis test. **e,** Analysis of histopathology in mice of indicated genotypes. Haematoxylin and eosin staining of sections of the indicated organs. Images are representative of 9 *Foxp3*^DTR-GFP/y^ and 7 *Foxp3*^LSL/y^ mice. **f-h,** Percentages of activated, proliferating, and cytokine-producing conventional CD4 T cells (**f**), CD8 T cells (**g**) and myeloid populations (**h**) from indicated organs. Data are pooled from two independent experiments. Two-tailed unpaired *t*-tests with Holm-Sidak multiple comparison. pLN, peripheral (brachial, axillary, and inguinal) lymph nodes; mLN, mesenteric lymph nodes; LP, lamina propria. All error bars denote mean ± s.e.m. ns, non-significant; *, p<0.05; **, p<0.01; ***, p<0.001; ****, p<0.0001.

**Figure 5 |:**
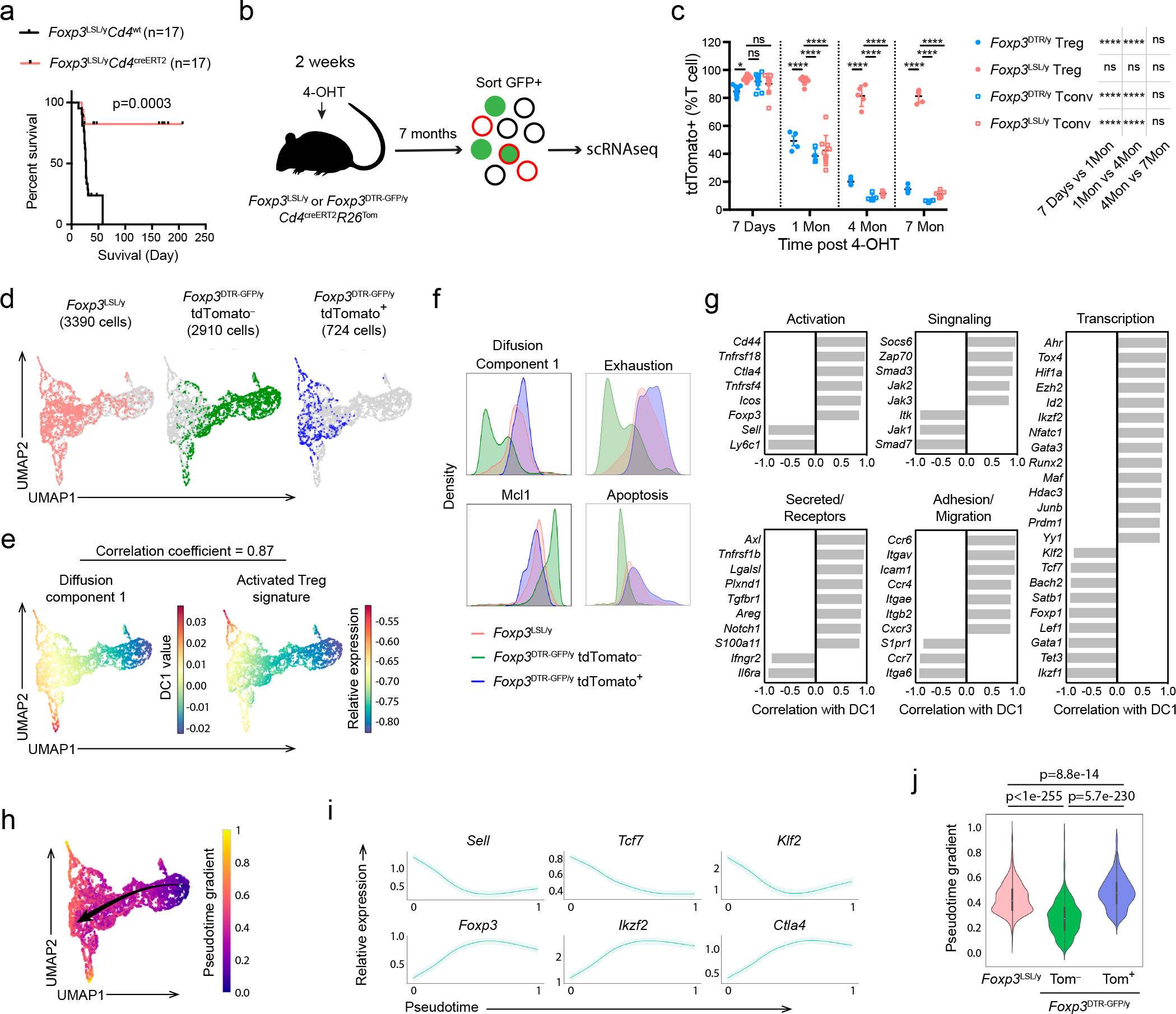
Single-cell transcriptomic analysis of control and long-lived rescued Treg cells. **a,** Survival plot of 4-OHT-treated male *Foxp3*^LSL^*Cd4*^wt^ and *Foxp3*^LSL^*Cd4*^creERT2^ mice. Mantel-Cox test. **b,** Experimental design. Mice were treated with 4-OHT at 2 weeks of age and Foxp3^+^ Treg cells, FACS-purified based on GFP expression, were subjected to scRNA-seq analysis using 10X Genomics platform 7 months later. **c,** Percentages of fate-mapped splenic Treg cells and conventional CD4 T cells in control and rescued mice at indicated time points post 4-OHT treatment. Scatter plot shows mean ± s.e.m. Two-way ANOVA with Tukey’s multiple comparison correction. **d,** UMAP visualization of the single-cell transcriptomes of experimental *Foxp3*^LSL^ and control tdTomato^−^ or tdTomato^+^ *Foxp3*^DTR-GFP^ Treg cells. **e,** UMAP visualization colored by diffusion component 1 (DC1, left) and the expression level of activated Treg cell gene signature (right). **f,** Histograms depicting the density of *Foxp3*^LSL^ and tdTomato^+^ or tdTomato^−^ *Foxp3*^DTR-GFP^ Treg cells along DC1, *Mcl1* expression, and the average expression levels of indicated gene sets. **g,** Correlation of curated genes with DC1. **h**, UMAP visualization colored by pseudotime generated with Palantir. Arrow indicates the direction of differentiation across the map. **i**, Expression of representative genes along the pseudotime trajectory. **j,** Violin plots showing the pseudotime values of *Foxp3*^LSL^ and tdTomato^+^ or tdTomato^−^ *Foxp3*^DTR-GFP^ Treg cells. White dots denote medians. Thick vertical bars delimit the interquartile ranges. Thin vertical lines represent upper and lower adjacent values. Histograms show distributions of the data, maxima and minima. Two-tailed *t*-test.

To test whether the containment of autoimmunity afforded by a single cohort of Treg cells was durable, we monitored tdTomato-labeled and -unlabeled cells in 4-OHT-treated male *Foxp3*^LSL^*Cd4*^creERT2^*R26*^Tom^ and control *Foxp3*^DTR-GFP^*Cd4*^creERT2^*R26*^Tom^ mice for an extended period of time (Fig. 4a). Within a month after treatment, the *Foxp3*^LSL^*Cd4*^creERT2^*R26*^Tom^ mice recovered completely from otherwise lethal disease and continued to gain weight (Extended Data Fig. 7a). At the 4-month time point, only recirculating Treg cells lacking CD73 expression were detected in the thymus, suggesting a lack of continuing thymic Treg output in agreement with our observation that *Cd4*^creERT2^-mediated recombination ceased within 48–72 hours after 4-OHT administration (Fig. 4b; Extended Data Fig. 7b)^34^. The peripheral “4-month-old” Treg pool was well maintained in both lymphoid and non-lymphoid tissues and remained functionally competent (Fig. 4c; Extended Data Fig. 7c). Nearly all Thy1.1^−^GFP^+^Foxp3^+^ Treg cells in *Foxp3*^LSL^*Cd4*^creERT2^*R26*^Tom^ mice expressed the tdTomato reporter, whereas Thy1.1^+^GFP^−^ “wannabes” and Thy1.1^−^GFP^−^Foxp3^−^ conventional CD4 T cells in the same mice, as well as Treg and conventional CD4 T cell subsets in control *Foxp3*^DTR-GFP^*Cd4*^creERT2^*R26*^Tom^ mice, contained only a small fraction of tdTomato^+^ fate-mapped cells (Fig. 4d). Thus, Treg cells generated as a single cohort in diseased *Foxp3*^LSL^*Cd4*^creERT2^ mice continued to persist while the other T cell subsets turned over. Impressively, even after 4 months, the reversal of autoimmune disease seemed complete with no tissue pathology observed and T cell activation, effector cytokine production, and myelo-proliferation, at best minimally increased (Fig. 4e–h; Extended Data Fig. 7d–f). Thus, a single cohort of Treg cells is capable of reversing established inflammation and affording long-term protection against autoimmunity. The rescued mice survived for at least 7 months (Fig. 5a). Even at this time point, the rescued mice remained largely healthy with only moderately increased T cell activation and effector cytokine production and well-maintained Treg populations in both lymphoid and non-lymphoid tissues (Extended Data Fig. 8a–c). Despite mildly elevated serum IgG1and IgE levels in comparison to the 4-week time point, the other Ig isotype levels remained comparable to those in control *Foxp3*^DTR-GFP/y^*Cd4*^creERT2^ mice (Extended Data Fig. 8d). Neither did we observe prominent immune infiltrates in the skin, liver, and small intestine (Extended Data Fig. 8e).

Since TCR specificity and diversity are essential for Treg-mediated control of autoimmunity^35, 36^, we assessed the TCR repertoires of long-lived redeemed Treg cells in *Foxp3*^LSL/y^ *Cd4*^creERT2^ mice by sequencing their TCRα chains at different time points after Foxp3 restoration. We observed a trend, albeit not statistically significant, toward a slight reduction in TCR diversity, as reflected in the gradually decreasing inverse Simpson indeces (Extended Data Fig. 8f). At later time points (5 and 7 months), the rescued Treg cells contained moderately reduced numbers of total unique clones compared to an earlier time point after the inflammatory disease subsided (1.5 months). The “unevenness” of the TCR repertoire measured by Gini coefficient remained unchanged throughout the entire time course (Extended Data Fig. 8g). These results suggested that the clonal diversity of long-lived redeemed Treg cells was largely preserved even though their TCR richness might have very mildly contracted. Thus, a cohort of restored Treg cells maintained a diverse TCR repertoire, which was likely essential for their ability to confer long-term containment of autoimmune inflammation.

### Transcriptional features of long-lived protective Treg cells

The extraordinarily long-term protection against autoimmunity by a single cohort of self-renewing Treg cells suggests that these cells do not exhibit dysfunction typical of chronically stimulated CD4 and CD8 T cells. Thus, we sought to analyze this long-lived Treg population at single-cell resolution using single cell RNA sequencing (scRNA-seq). We compared GFP^+^ Treg cells from *Foxp3*^LSL/y^*CD4*^creERT2^*R26*^Tom^ mice and *Foxp3*^DTR-GFP/y^*CD4*^creERT2^*R26*^Tom^ control mice 7 months after 4-OHT treatment (Fig. 5b). Treg cells in experimental and control mice were efficiently and comparably labeled by tdTomato shortly after 4-OHT administration (Fig. 5c). The percentage of tdTomato^+^ cells among Thy1.1^−^GFP^+^ Treg cells in *Foxp3*^LSL/y^*Cd4*^creERT2^*R26*^Tom^ mice remained high (>80%), demonstrating their impressive persistence. However, among control *Foxp3*^DTR-GFP^ Treg cells, tdTomato positivity gradually declined to ~15% at 7 months, most likely due to continuous thymic output of tdTomato^−^ Treg cells (Fig. 5c).

**Extended Data Figure 9 |:**
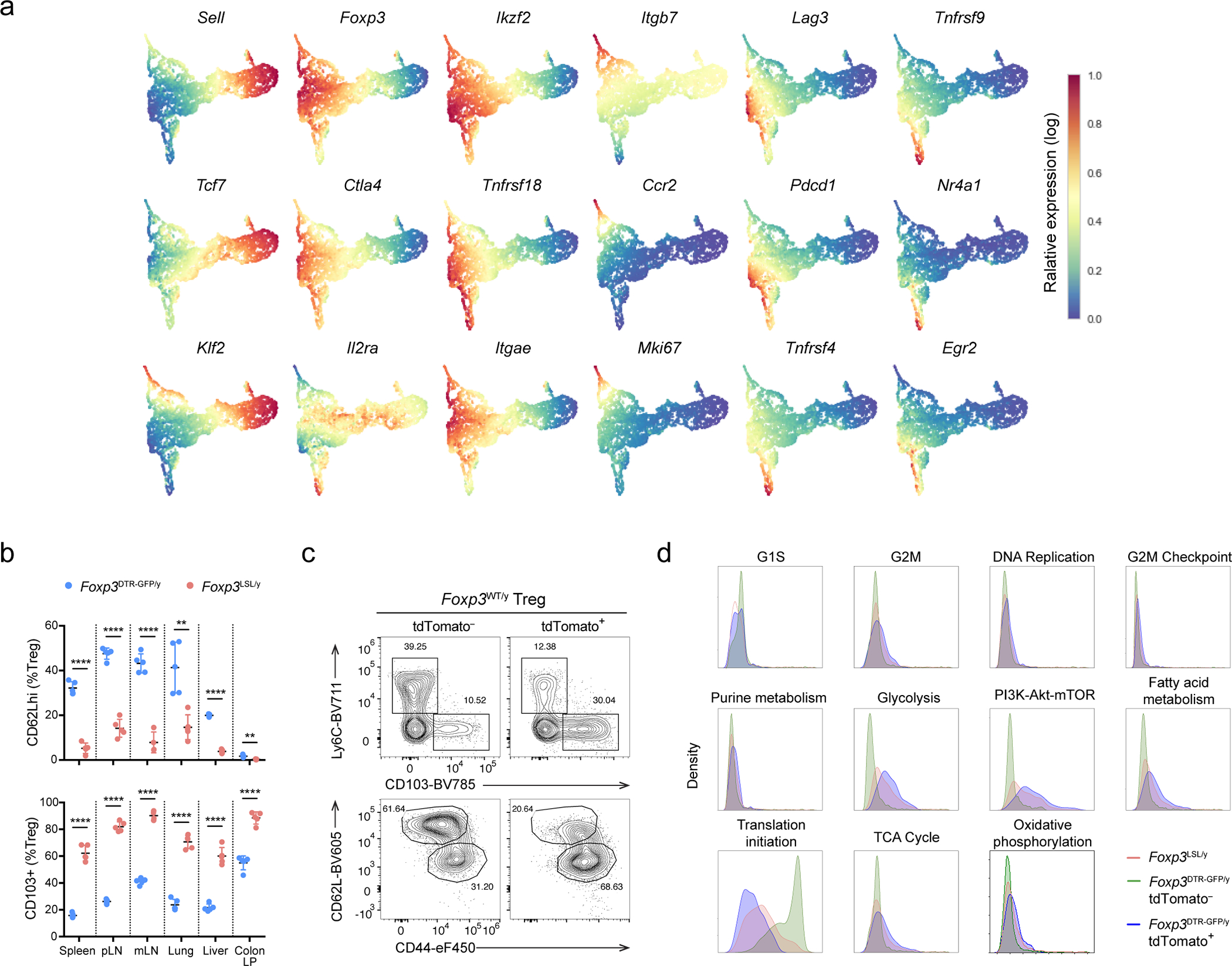
Analysis of long-lived Treg cells in *Foxp3*^LSL^ and control *Foxp3*^DTR-GFP^ mice. Mice were treated with 4-OHT on postnatal day 14 and analyzed 7 months later. **a,** UMAP visualization of the single-cell transcriptomes colored by imputed expression levels of representative genes. **b**, Frequencies of CD62L^hi^ and CD103^+^ cells among Treg cells in rescued *Foxp3*^LSL^*Cd4*^creERT2^ and control *Foxp3*^DTR-GFP^*Cd4*^creERT2^ mice. Two-tailed multiple *t*-tests. pLN, peripheral (brachial, axillary, and inguinal) lymph nodes; mLN, mesenteric lymph nodes; LP, lamina propria. **c,** *Foxp3*^WT^*Cd4*^creERT2^*R26*^Tom^ mice were treated with 4-OHT on postnatal day 14 and analyzed 4 months later. Representative contour plots show expression of activation markers by tdTomato^+^ and tdTomato^−^ Foxp3^+^ CD4 T cells. **d,** Histogram depicting the density of *Foxp3*^LSL^ and tdTomato^+^ or tdTomato^−^ *Foxp3*^DTR-GFP^ Treg cells along the average expression values for the indicated gene sets. All error bars denote mean ± s.e.m. ns, non-significant; *, p<0.05; **, p<0.01; ***, p<0.001; ****, p<0.0001.

In the scRNA-seq data analysis, we distinguished “old” and “young” control Treg cells from *Foxp3*^DTR-GFP/y^*Cd4*^creERT2^*R26*^Tom^ mice by computationally separating them into tdTomato-positive (“old”) and -negative (“young”) cells based on the expression of tdTomato transcript by the individual cells. Thy1.1^−^GFP^+^ Treg cells in *Foxp3*^LSL/y^*Cd4*^creERT2^*R26*^Tom^ mice, as well as tdTomato^+^ and tdTomato^−^ control GFP^+^ Treg cells in *Foxp3*^DTR-GFP/y^*Cd4*^creERT2^*R26*^Tom^ mice, exhibited distinct UMAP distribution patterns (Fig. 5d). To better understand the most significant sources of variation among the three Treg cell populations, we performed diffusion map analysis. The first diffusion component (DC1) had a highly similar distribution of gene expression to that of activated Treg gene signature^30^ (Fig. 5e). The long-lived Thy1.1^−^GFP^+^ Treg cells had a DC1 density resembling that of tdTomato^+^ control Treg cells, both higher than that of tdTomato^−^ cells (Fig. 5f). This result indicates that long-lived Treg cells in *Foxp3*^LSL/y^*Cd4*^creERT2^*R26*^Tom^ and control mice tagged with tdTomato 7 month earlier and their offspring exhibited a similarly activated phenotype. Upon examining genes whose expression patterns highly correlated with DC1, we found transcripts associated with Treg activation (*Cd44*, *Sell*, *Tnfrsf18*, *Icos*, *Ctla4*), migration to non-lymphoid tissues (*Ccr4*, *Cxcr3*, *Ccr6, Itgae*), as well as tissue adaptation and suppressor function (*Maf*, *Ahr*) (Fig. 5g, Extended Data Fig. 9a)^37, 38^. Flow cytometric analysis of CD62L (*Sell*) and CD103 (*Itgae*) expression confirmed the scRNA-seq results across multiple tissues, showing sharp reduction in CD62L^hi^ and increase in CD103^+^ cells among Treg cells in *Foxp3*^LSL/y^*Cd4*^creERT2^*R26*^Tom^ mice (Extended Data Fig. 9b). Furthermore, the tdTomato^+^ fraction of control Treg cells were enriched for Ly6C^−^CD103^+^ and CD44^high^CD62L^lo^ cells, consistent with their heightened activation state (Extended Data Fig. 9c). Despite the burden of being the sole Treg population responsible for immunosuppression, the Thy1.1^−^GFP^+^ Treg cells did not appear less fit than their “age-matched” counterparts in control mice with continuous Treg turnover. Compared to tdTomato^+^ Treg cells in *Foxp3*^DTR-GFP/y^*Cd4*^creERT2^*R26*^Tom^ mice, Thy1.1^−^GFP^+^ Treg cells in *Foxp3*^LSL/y^*Cd4*^creERT2^*R26*^Tom^ mice exhibited comparable expression of genes associated with T cell exhaustion^39^ and apoptosis, and similar levels of *Mcl1*, an anti-apoptotic gene critical for Treg survival^40^ (Fig. 5f). Genes involved in cell cycle progression and various metabolic pathways were also comparably expressed by tdTomato^+^ Treg cells in *Foxp3*^DTR-GFP/y^*Cd4*^creERT2^*R26*^Tom^ mice and Thy1.1^−^GFP^+^ Treg cells in *Foxp3*^LSL/y^*Cd4*^creERT2^*R26*^Tom^ mice (Extended Data Fig. 9d). These shared transcriptional features highlighted the similarities in metabolic status and proliferative potential between the long-lived Treg cells in *Foxp3*^LSL/y^*Cd4*^creERT2^*R26*^Tom^ mice and “age-matched” tdTomato^+^ control *Foxp3*^DTR-GFP/y^ Treg cells.

To further compare Thy1.1^−^GFP^+^ Treg cells in *Foxp3*^LSL/y^*Cd4*^creERT2^*R26*^Tom^ mice and long-lived control cells present within the peripheral Treg pool under physiologic conditions, we performed trajectory analysis using the Palantir algorithm, which generates a high-resolution pseudotime ordering of cells at different developmental stages^41^. The pseudotime gradient started from resting Treg cells, proceeded towards activated Treg cells, and ended with cells reminiscent of Treg cells residing in non-lymphoid organs in agreement with a previous report^42^ (Fig. 5h). The analysis successfully recapitulated known gene expression dynamics during Treg activation including downregulation of *Tcf7*, *Sell*, and *Klf2,* and upregulation of *Foxp3*, *Ctla4*, and *Ikzf2* (Fig. 5i). Next, we compared the pseudotemporal ordering of Thy1.1^−^GFP^+^ Treg cells from *Foxp3*^LSL/y^*Cd4*^creERT2^*R26*^Tom^ mice derived from cells whose Foxp3 expression was “restored” 7 months ago, with that of similarly “old” tdTomato^+^ and of “young” tdTomato^−^ Treg cells from control *Foxp3*^DTR-GFP/y^*Cd4*^creERT2^*R26*^Tom^ mice. Both “old” Treg populations were positioned late in the inferred differentiation trajectory in comparison to tdTomato^−^ Treg cells—consistent with their activated phenotype—and were characterized by comparablely high pseudotime values (Fig. 5j). Thus, Thy1.1^−^GFP^+^ Treg cells in *Foxp3*^LSL/y^*Cd4*^creERT2^*R26*^Tom^ mice closely resembled a subset of long-lived cells present in the normal Treg cell pool under physiologic conditions.

**Extended Data Figure 10 |:**
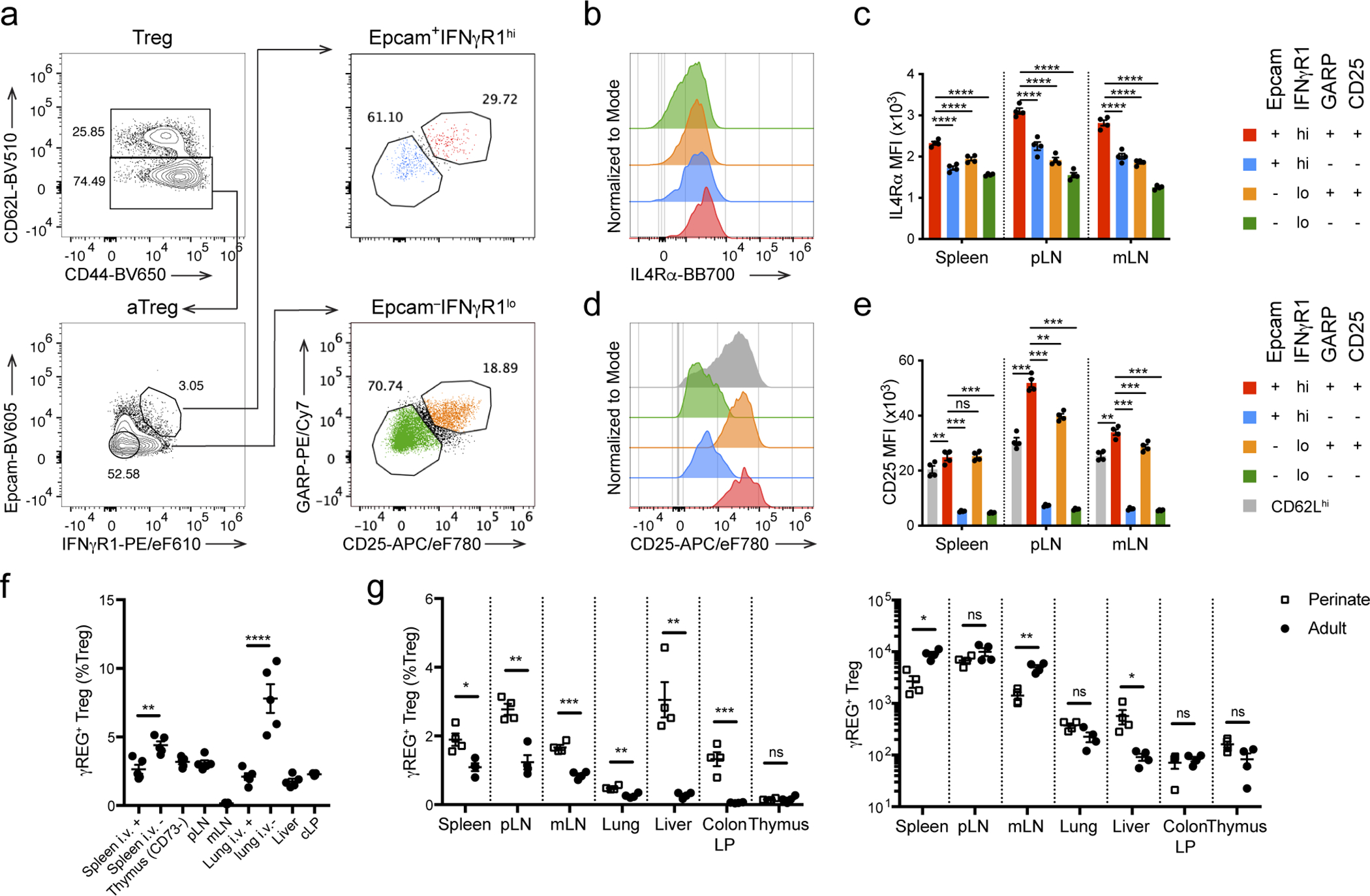
Flow cytometric analysis of γREG^+^ Treg cells. **a,** Gating strategy for γREG^+^ Treg cells in adult mice. **b, c,** Representative histogram (**b**) and quantification (**c**) of IL-4Rα expression in γREG^+^ and other Treg cell populations identified in **a**. Two-way ANOVA with Tukey’s multiple comparison correction. **d, e,** Representative histogram (**d**) and quantification (**e**) of CD25 expression in γREG^+^ and other Treg cell populations identified in **a**. Two-way ANOVA with Tukey’s multiple comparison correction. **f,** Percentages of γREG^+^ Treg cells in lymphoid organs and non-lymphoid tissues of CD45 i.v.-labeled 8–10-week-old *Foxp3*^*GFP*^ mice. One-way ANOVA with Sidak’s multiple comparison test. **g,** Percentages (left) and numbers (right) of γREG^+^ Treg cells in different organs of 2- or 8-week-old *Foxp3*^*GFP-BirA-AVI-TEV*^ mice. Two-sided unpaired *t*-tests with correction for multiple hypothesis testing using the Holm-Sidak method. All error bars denote mean ± s.e.m. ns, non-significant; *, p<0.05; **, p<0.01; ***, p<0.001; ****, p<0.0001. pLN, peripheral (brachial, axillary, and inguinal) lymph nodes; mLN, mesenteric lymph nodes; cLP, colonic lamina propria.

**Figure 6 |:**
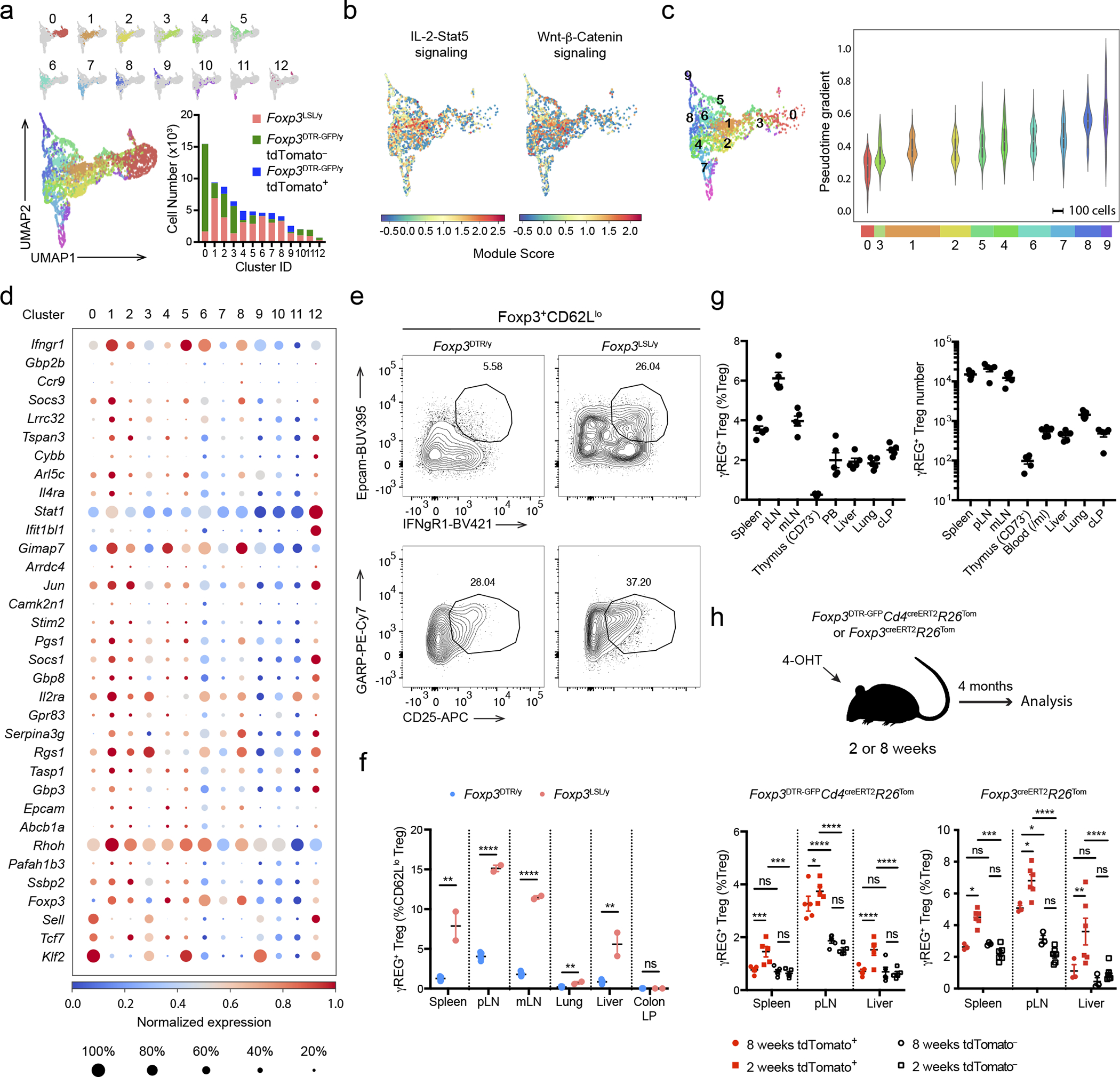
Identification and analysis of γREG^+^ Treg cells from the single-cell RNA-seq data. **a,** UMAP visualization colored by the clusters. Bar graph shows the numbers of cells from each sample that contributed to the individual clusters. **b**, UMAP visualization of *Foxp3*^LSL^ Treg cells colored by the Seurat module scores for the indicated gene sets. **c**, Violin plots showing the pseudotime values of *Foxp3*^LSL^ Treg cells from the top 10 clusters. White dots denote medians. Thick vertical bars delimit the interquartile ranges. Thin vertical lines represent upper and lower adjacent values. Histograms show distributions of the data, maxima and minima. Horizontal bars indicate the numbers of *Foxp3*^LSL^ Treg cells contributing to each cluster. **d**, Dot plot showing the expression of top enriched genes and underrepresented genes (p<0.05) from cluster 1 expressed by more than 20% of the cells in at least one cluster. P-values for enriched genes were calculated with two-tailed *t*-test, as used by the default parameters of Scanpy. See Supplementary Table 1 for list of enriched genes by cluster. **e-f,** Representative plots (**e**) and combined data (**f**) showing the frequencies of γREG^+^ Treg cells among splenic CD62L^lo^ Treg cells in rescued and control mice treated with 4-OHT on postnatal day 14 and analyzed 4 months later. Two-tailed unpaired *t*-tests with Holm-Sidak multiple comparison. **g**, Frequencies (left) and absolute numbers (right) of γREG^+^ Treg cells in various tissues of unmanipulated 8-week-old *Foxp3*^GFP^ mice. **h**, Percentages of γREG^+^ Treg cells among tdTomato^+^ or tdTomato^−^ Treg cells “time-stamped” in adult (8-week-old) or perinatal (2-week-old) mice and analyzed after 4 months. Two-way ANOVA with Tukey’s multiple comparison test. All error bars denote mean ± s.e.m. ns, non-significant; *, p<0.05; **, p<0.01; ***, p<0.001; ****, p<0.0001. pLN, peripheral (brachial, axillary, and inguinal) lymph nodes; mLN, mesenteric lymph nodes; PB, peripheral blood; cLP, colonic lamina propria.

To gain further insights into the long-term persistence of Thy1.1^−^GFP^+^ Treg cells in *Foxp3*^LSL^*Cd4*^creERT2^*R26*^Tom^ mice, we performed a refined clustering analysis of the scRNA-seq dataset. Among the 13 clusters identified, clusters 0 and 3 were primarily composed of tdTomato^−^ *Foxp3*^DTR-GFP^ control Treg cells, whereas clusters 1, 6, 7 and 8 were enriched for Thy1.1^−^GFP^+^ Treg cells (Fig. 6a). Importantly, cells from cluster 1 had higher expression of IL-2–Stat5 and Wnt–β-catenin pathway gene signatures (Fig. 6b), which have been implicated in Treg cell maintenance and self-renewal^43, 44, 45^. Moreover, these cells were lower in pseudotime values compared to most Thy1.1^−^GFP^+^ Treg cells, suggesting that they were less differentiated and had the potential to give rise to the rest of the cells and sustain the Treg pool (Fig. 6c). Gene expression analysis revealed transcripts enriched (*Ifngr1*, *Epcam*, *Lrrc32*, *Il4ra, etc.*) and depleted (*Sell*, etc.) in cluster 1 cells (Fig. 6d, Supplementary Table 1). Using flow cytometry, we corroborated these results by demonstrating a marked enrichment of an IL-4Rα^hi^IFNγR1^hi^Epcam^+^GARP^hi^CD25^hi^ (γREG^+^) cell subset within activated CD62L^lo^ rescued Treg population in *Foxp3*^LSL^*Cd4*^creERT2^*R26*^Tom^ mice (Fig. 6e–f, Extended Data Fig. 10a–e). In normal mice, these cells were also present, albeit at lower frequencies, within Treg population in secondary lymphoid organs and at even lower frequencies in non-lymphoid tissues, such as the liver and lung, but were virtually undetectable among newly differentiated CD73^−^ thymic Treg cells (Fig. 6g). γREG^+^ Treg cells were enriched in the parenchyma of highly vascularized organs such as spleen and lung, as evidenced by their higher frequencies among cells not labeled by intravenously administered CD45 antibody. This suggested that these cells were largely non-circulatory and likely contributed to local maintenance of the Treg pools (Extended Data Fig. 10f). To compare the capacity of perinatal and adult Treg populations to give rise to γREG^+^ Treg cells, we labeled Treg cells in healthy 2-week-old and 8-week-old *Foxp3*^DTR-GFP^*Cd4*^creERT2^*R26*^Tom^ mice by 4-OHT treatment and assessed the frequencies of γREG^+^ Treg cells within tdTomato^+^ and tdTomato^−^ Treg subsets 4 months later. In mice treated as perinates, tdTomato^+^ Treg cells harbored higher frequencies of γREG^+^ Treg cells than their tdTomato^−^ counterparts (Fig. 6h). In contrast, tdTomato^+^ Treg cells time-stamped during adulthood were not enriched for γREG^+^ Treg cells over their tdTomato^−^ counterpart, and similar percentages of γREG^+^ cells were found among tdTomato^−^ Treg cells in perinatally labeled mice (Fig. 6h). These observations were independently confirmed by similar time-stamping of Treg cells in young vs. adult *Foxp3*^creERT2^*R26*^Tom^ mice (Fig. 6h). These results were consistent with the enrichment of γREG^+^ Treg cells among Treg populations in secondary lymphoid organs and non-lymphoid tissues in unmanipulated 2-week-old vs. 8-week-old mice (Extended Data Fig. 10g). Our results suggest γREG^+^ Treg cells, capable of persisting in peripheral lymphoid and non-lymphoid organs, are selectively overrepresented within the Treg population generated early in life.

## Discussion

The adaptive immune system is characterized by unlimited antigen recognition specificity, amplification of innate immune responses, novel effector functions, and memory formation, affording vertebrates with a versatile anticipatory defense against rapidly evolving infectious agents. This superior protection is confounded by a major fitness constraint due to the threat of autoimmune inflammation posed by the inherent self-reactivity of T cells. The importance of Treg cells in forestalling autoimmunity has been most vividly demonstrated by wide-ranging clinical manifestations of human monogenic disorders resulting from Treg deficiency or dysfunction due to *FOXP3*, *STAT5B, IL2RA,* and *LRBA* mutations or *CTLA4* haplo-insufficiency^46, 47^. In IPEX patients, these manifestations include endocrinopathies (diabetes, thyroiditis, pancreatitis, adrenal dysfunction), hepatitis, enteropathies (autoimmune gastritis, IBD, celiac disease), skin disorders (exudative dermatitis, alopecia), food allergy, hyper-IgE syndrome, autoimmune hematologic disorders (autoimmune thrombocytopenia, hemolytic anemia), myelo- and lymphoproliferation (splenomegaly, lymphadenopathy), polyneuropathy, as well as pulmonary and nephrotic syndromes^48^. In mice, Treg deficiency or depletion causes equally widespread and fatal autoimmune inflammatory disease^13^.

However, the potent immunosuppressive function of Treg cells indispensable for preventing autoimmune inflammation can in turn compromise protective immunity against infections. This provided rationale for the concept that Treg cells are ineffectual in the inflammatory settings of infections. Indeed, numerous studies showed that infection-associated inflammatory milieu can cause downregulation of Foxp3 expression by Treg cells, impede their suppressor and proliferative capacity, or even confer pro-inflammatory properties, i.e. effector cytokine production; furthermore, pro-inflammatory cytokines, IL-6 and IL-1 in particular, can make effector cells refractory to Treg-mediated suppression^14, 16, 18, 19, 49, 50^. On the other hand, some studies suggested that Treg cells are capable of modulating virus-specific immune responses and limiting associated pathologies^51, 52, 53, 54, 55, 56^. In all these studies, however, Treg cells were present from the onset of infection, making it impossible to discriminate their activity prior to, or after the onset of infection-induced inflammation. Thus, the ability of Treg cells to function in infection-induced inflammation remains an open question.

Since inflammation elicited by various infectious, metabolic, and genetic causes, including Treg deficiency, shares principal cellular and soluble mediators, a corollary to the above concept is the notion that in established autoimmune inflammation, Treg activity is also expected to be severely attenuated or even lacking. In this regard, autoimmune and inflammatory pathologies were reported to exert varying effects on Treg numbers. In contrast to the aforementioned monogenic Treg deficiency-linked disorders, many studies of polygenic autoimmune diseases have found unchanged or even increased frequencies of Treg cells at inflammatory sites and in circulation^57^. Meanwhile, analyses of Treg cells isolated from autoimmune patients suggested that their function is impaired in the inflammatory environment^21, 22, 23, 57, 58, 59^. However, considering numerous genetic polymorphisms linked to the pathophysiological manifestations of polygenic autoimmune diseases, the reported inferior Treg functionality can be one of these sequelae. Indeed, an enrichment for such autoimmunity-associated single nucleotide polymorphisms has been observed within Treg-specific cis-regulatory elements^10, 60, 61^. As a potential counterargument to the above findings, adoptive transfer of Treg cells after the initiation of T cell-induced colitis was shown to cause its amelioration over time in an IL-10-dependent manner, highlighting their potential to control local inflammation elicited upon homeostatic expansion of a relatively small number of naïve CD4 T cells in lymphopenic mice^62, 63^. Thus, it remained possible that the reparative activity of Treg cells observed in this setting may be specific to the colon aided by its continuous epithelial cell turnover, or particular to the adoptive transfer model.

By genetically introducing a limited Treg pool in *Foxp3*^LSL^ mice, we demonstrated that Treg cells are able to function in settings of systemic autoimmune inflammation, reverse the fatal disease and tissue pathology, and offer long-term protection. The reversal of inflammation was not a simple diversion of a large number of pro-inflammatory effector T cells into Treg cells because indistinguishable fatal autoimmunity observed in Foxp3-deficient mice harboring Treg “wannabes” and *Foxp3*^*DTR*^ neonates subjected to chronic ablation of Treg cells suggests that Treg “wannabes” do not provide notable non-redundant contribution to the disease progression^5, 8, 13^. It is noteworthy that upon generation of functional Treg cells in *Foxp3*^LSL^ mice, the reduction in myeloid cells was observed with a markedly slower kinetics than the decrease in activated T cell numbers and responses, suggesting that the reversal of myelo-proliferation was largely an indirect effect of suppression of T cell activation. Nevertheless, a parallel direct suppression of myeloid cell activation and survival by Treg cells may also contribute to the recovery of *Foxp3*^LSL^ mice from autoimmunity. The ability of Treg cells to suppress established inflammation was not limited to young mice but was also observed in adult animals. Notably, Treg cells introduced into the inflammatory environment suppressed Th2 autoimmunity and associated increased serum IgG1 and IgE levels, as efficiently as Th1 and Th17 responses, even though Th2 responses are known to be the most sensitive to diminished Treg numbers or functionality^26, 64^.

The observed normalization of lympho- and myeloproliferation, acute phase response, and reversal of tissue inflammation highlighted the ability of Treg cells to function in a cytokine storm. Rather than impeding functionality, we found that exposure to this highly inflammatory milieu endowed the newly generated Treg cells with heightened suppressor activity in comparison to Treg cells from healthy control mice, while the latter and Treg cells generated upon a similar conversion of Treg “wannabes” in healthy heterozygous female mice had indistinguishable suppressor activity. Treg cells employ multiple and partially redundant modes of suppression, including secreted immunomodulatory factors, IL-2 consumption, and ATP-to-adenosine conversion, and may directly partake in tissue regeneration and repair via the production of amphiregulin^65, 66^. Accordingly, redeemed Treg cells in *Foxp3*^LSL^ mice exhibited increased expression of IL-2R, IL-10, galectin-1 and –3, Fgl2, CTLA4, CD39, ST2, and amphiregulin. While we cannot unambiguously pinpoint the inflammation-sensing pathways responsible for the potentiation of their function, prominent STAT5 activation gene signature in these cells implicates common γ-chain receptor signaling cytokines including IL-2. Accordingly, administration of therapeutic IL-2 formulations and expression of an active form of STAT5b in Treg cells promote their expansion and superior suppressor function^31, 67, 68^. Notably, the observed suppressor activity of Treg cells in inflammatory settings was not limited to recent thymic emigrants as efficient suppression of autoimmunity in systemically inflamed mice was observed upon pharmacological thymic export blockade. The reversal of inflammatory disease and long-term maintenance of health by a single pool of Treg cells was enabled by its stability, which may be aided by a subset of preferentially tissue-residing cells with increased expression of IFNγR, IL-4Rα, EpCAM, CD25, and GARP. Based on gene set enrichment and pseudo-time analyses, these cells may contribute to long-term Treg maintenance by giving rise to terminally differentiated progenies locally. Interestingly, time-stamped “old” peripheral Treg population in lymph nodes of adult mice was enriched for γREG^+^ Treg cells in comparison to other sites such as the spleen or liver, raising the possibility of specific niches that favor their generation or maintenance.

Collectively, we show that Treg cells can function in established severe inflammation and reverse all known types of inflammation, and that a single cohort of Treg cells can afford long-lasting protection against autoimmunity. Our findings provide general rationale and support for the emerging efforts to develop adoptive Treg therapy not only for intrauterine and neonatal IPEX syndrome and other monogenic Treg deficiencies, but also for a broad spectrum of autoimmune and inflammatory disorders.

## Methods

### Mice

Experiments in this study were approved by the Sloan Kettering Institute (SKI) Institutional Animal Care and Use Committee under protocol 08–10-023 and conducted in compliance with institutional ethics guidelines. Mice were housed at the SKI animal facility under SPF conditions on a 12-hour light/dark cycle with free access to water and regular chow diet. The average ambient temperature is 21.5°C and the average humidity is 48%. All control and experimental animals were age-matched, and littermates were used as controls unless otherwise indicated. Age, sex, and numbers of animals used in each experiment are indicated in the respective Figure Legends.

*Foxp3*^DTR-GFP 13^, *Foxp3*^GFP 6^, *Foxp3*^creERT2 28^,*Foxp3*^GFP-BirA-AVI-TEV/y^ (in preparation), *Cd4*^creERT2 69^, *Tcrb*^*−/−*^*Tcrd*^*−/−* 70^ and *Rosa26*^LSL-tdTomato 71^ mice were maintained in the the Sloan Kettering Institute animal facility. The *Rosa26*^LSL-tdTomato^ allele was bred to homozygosity for fate mapping studies.

### Generation of *Foxp3*^LSL^ mice

The targeting construct was made by first subcloning an 8.5 kb *SphI* fragment harboring the *Foxp3* genomic sequence from exon -1a to exon 7 obtained from the RP23–143D8 cosmid into a cloning vector carrying a Pgk-DTA-polyA cassette allowing for negative selection of random genomic integration. The FRT-PGK-neo-polyA-FRT positive selection cassette was then cloned into the *DraIII* site within the intron between exons -1b and 1, and the loxP-Thy1.1 coding sequence-triple-tandem SV40 polyA-eGFP coding sequence cassette was cloned into the *AvrII* site within exon 1. The Thy1.1 and eGFP sequences are preceded by a start codon (ATG). The targeting construct was then electroporated into albino C57BL/6 ES cells. After neomycin selection, Southern blotting and karyotyping, correctly targeted clones were injected into WT C57BL/6 blastocysts. The resulting chimeric mice were bred to albino C57BL/6 mice. Founders identified based on the coat color and genotyping were mated to a Flp deleter strain of mice to remove the neo cassette.

### 4-hydroxytamoxifen, diphtheria toxin, tamoxifen, and FTY720 treatment

4-hydroxytamoxifen (4-OHT, Sigma-Aldrich, H7904) stock solution was made by reconstituting in ethyl alcohol 200 proof (Sigma-Aldrich, E-7023) at a concentration of 20 mg/mL, then diluting 1:1 (v/v) with Cremophor EL (Sigma-Aldrich, 238470). 20 μg/g body weight of 4-OHT diluted in PBS was administered intraperitoneally.

Diphtheria toxin (DT, List Biological Laboratories, 150) was dissolved in PBS. 1μg (first dose) or 250 ng (subsequent doses) was injected intraperitoneally for each mouse. For tamoxifen administration, 20 mg of tamoxifen (Sigma-Aldrich, T5648) was resuspended in 1 mL corn oil (Sigma-Aldrich, C8267) by rotating and tilting at 37°C until fully dissolved. Each mouse was orally gavaged with 4 mg of tamoxifen per treatment.

4-OHT was used whenever possible because it bypasses the conversion of tamoxifen to 4-hydroxytamoxifen in the liver. Compared to tamoxifen, 4-OHT offers a much sharper pharmacokinetics and enables a highly synchronized labeling of cells. In addition, 4-OHT has a much shorter half-life than tamoxifen, particularly in adults. Therefore, tamoxifen instead of 4-OHT was used for *in vivo* suppression assays (Extended Data Fig. 1g) and in studies of adult female mice (Fig. 2, Fig. 3 h–j, and Extended Data Fig. 5) to achieve sufficient recombination of the *Foxp3*^LSL^ allele.

FTY720 (Sigma-Aldrich, SML0700–5MG) stock solution was made by reconstituting in dimethyl sulfoxide (Sigma-Aldrich, D8418–250ML) at a concentration of 20 mg/ml. 0.8 μg/g body weight of FTY720 diluted to 0.1 mg/ml in 2% β-hydroxypropanylcyclodextrin (Sigma-Aldrich, H5784–10ML) was administered intraperitoneally.

### Reagents and antibodies

The following antibodies and streptavidin were used in this study for flow cytometry, with clones, venders, catalog numbers, and dilutions indicated in the parentheses: anti-Siglec-F (E50-2440, BD, 562681, 400), anti-I-A/I-E (M5/114.15.2, BioLegend, 107641, 1000), anti-NK1.1 (PK136, ThermoFisher, 47-5941-82, 200), anti-CD45 (30-F11, BioLegend, 103136, 600), anti-CD11b (M1/70, BioLegend, 101257, 800), anti-CD3ε (17A2, BioLegend, 100237, 500), anti-Ly-6C (HK1.4, BioLegend, 128037, 1000), anti-CD90.2 (30-H12, BioLegend, 105331, 1000), anti-Foxp3 (FJK-16s, ThermoFisher, 17-5773-82, 400), anti-CD19 (6D5, BD, 563557, 400), anti-Ly-6G (1A8, BioLegend, 127618, 500), anti-TCRβ (H57-597, ThermoFisher, 61-5961-82, 400), anti-F4|80 (BM8, ThermoFisher, 61-4801-82, 200), anti-CD90.1 (HIS51, ThermoFisher, 17-0900-82, 200), anti-CD4 (RM4-5, BioLegend, 100553, 400), anti-CD8α (53-6.7, BioLegend, 100759, 400), anti-GITR (DTA-1, ThermoFisher, 48-5874-82, 400), anti-CD73 (TY/11.8, BioLegend, 127215, 400), anti-CD44 (IM7, BD, 563971, 400; ThermoFisher, 48-0441-82, 400; BioLegend, 103049, 400), anti-CD103 (M290, BD, 564322, 200), anti-CD62L (MEL-14, BioLegend, 104438, 1600; BioLegend, 104441, 400), anti-CTLA4 (UC10-4B9, BioLegend, 106314, 600), anti-Helios (22F6, BioLegend, 137218, 400), anti-Ki-67 (16A8, BioLegend, 652420, 400), anti-CD25 (PC61.5, ThermoFisher, 47-0251-82, 400; ThermoFisher, 17-0251-82, 400), anti-PD-1 (29F.1A12, BioLegend, 135225, 400), anti-ICOS (7E.17G9, ThermoFisher, 12-9942-82, 400), anti-CD45.1 (A20, BioLegend, 110738, 100), anti-CD45.2 (104, BD, 612778, 200), anti-IL-2 (JES6-5H4, BioLegend, 503818, 400), anti-IL-17A (17B7, ThermoFisher, 48-7177-82), anti-IFNγ (XMG1.2, ThermoFisher, 17-7311-82, 500), anti-IL-4 (BVD6-24G2, ThermoFisher, 25-7042-82, 400), anti-GARP (YGIC86, ThermoFisher, 25-9891-82, 200), anti-CD119 (2E2, BD, 550482, 100; GR20, BD, 740032, 100), anti-EpCAM (G8.8, BioLegend, 118227, 100; BD, 740281, 100), anti-CD124 (mIL4R-M1, BD, 742172, 100), anti-TCR Vβ5.1, 5.2 (MR9-4, BioLegend, 139508, 400), anti-TCR Vβ6 (RR4-7, ThermoFisher, 46-5795-82, 300), anti-TCR Vβ8 (F23.1, BD, 553860, 400), streptavidin (BioLegend, 405225, 4000; ThermoFisher, 61-4317-82, 4000), anti-CD45R/B220 (RA3-6B2, ThermoFisher, 47-0452-82, 400).

The following antibodies were used to capture antigens for ELISA in this study: purified anti-mouse IgE (R35-72, BD Pharmingen, 553413), Goat Anti-Mouse IgG1 (RRID: AB_2794408, SouthernBiotech, 1070-01), Goat Anti-Mouse IgG3 (RRID: AB_2794567, SouthernBiotech, 1100-01), Goat Anti-Mouse IgG2a (RRID: AB_2794475, SouthernBiotech, 1080-01), Goat Anti-Mouse IgG2b (RRID: AB_2794517, SouthernBiotech, 1090-01), Goat Anti-Mouse IgG2c (RRID: AB_2794464, SouthernBiotech, 1079-01), Goat Anti-Mouse IgA (RRID: AB_2314669, SouthernBiotech, 1040-01), Goat Anti-Mouse IgM (RRID: AB_2794197, SouthernBiotech, 1020-01), Mouse Pentraxin 2/SAP Antibody (R & D Systems, MAB2558).

The following antibodies were used to detect antigens for ELISA in this study: Goat Anti-Mouse Ig-HRP (RRID: AB_2728714, SouthernBiotech, 1010-05), Biotin Rat Anti-Mouse IgE (R35-118, BD Pharmingen, 553419), Biotinylated Pentraxin 2/SAP Antibody (R & D Systems, BAF2558).

The following reagents were used to construct standard curves for ELISA in this study: Purified Mouse IgE, kappa, Isotype Control (C38-2, BD Pharmingen, 557079), Purified Mouse IgA, kappa, Isotype Control (M18-254, BD Pharmingen, 553476), Purified Mouse IgG3, kappa, Isotype Control (A112-3, BD Pharmingen, 553486), Purified Mouse IgG1, kappa, Isotype Control (15H6, SouthernBiotech, 0102-01), Purified Mouse IgG2a, kappa, Isotype Control (UPC-10, Sigma, M5409), IgM Isotype Control from murine myeloma (MOPC 104E, Sigma, M5909), IgG2b Isotype Control from murine myeloma (MOPC-141, Sigma, M5534), Mouse IgG2c (6.3, RRID: AB_2794064, SouthernBiotech, 0122-01), Recombinant Mouse Pentraxin 2 (R & D Systems, 2558-SA-050).

### Enzyme-linked immunosorbent assay (ELISA)

ELISA experiments were conducted as previously described^36^. Briefly, mouse peripheral blood was collected via cardiac puncture immediately after euthanasia into BD SST microcontainer tubes (02-675-185) and sera were harvested after centrifugation. Flat-bottom 96-well plates were coated with capturing antibodies in 50 μL 0.1 M NaHCO_3_ solution at pH 9.5 O/N at 4°C. The plates were then emptied, blocked with 200 μL 1% bovine serum albumin (VWR, 97061-422) in PBS, and washed 3 times with PBS containing 0.05% Tween-20 (Sigma-Aldrich, P1379). 50 μL of sera at appropriate dilutions was added and incubated O/N at 4°C. The plate was then incubated in sequential orders with 50 μL of biotinylated detection antibodies for 2–3 hours, 50 μL of avidin-HRP (ThermoFisher, 18-4100-51) for 30 minutes, and 100 μL of TMB solution (ThermoFisher, 00-4201-56) at 25°C, with 3–4 washes with PBS-Tween in between each incubation steps. The colorimetric reaction was stopped with 100 μL of 1M H_3_PO_4_ (Sigma-Aldrich, P5811) after 5–10 minutes of adding TMB and absorbance at 450 nm was measured with a Synergy HTX plate reader (BioTek). Concentrations of antigens were determined using standard curves constructed with purified recombinant proteins and calculated with Gen5 3.02.2 (BioTek).

### Isolation of cells from lymphoid organs, livers, lungs, and colonic lamina propria

For flow cytometric analyses, animals were perfused with a total of 20 mL PBS into both left and right ventricles immediately after euthanasia. Cells were retrieved from spleens, peripheral (brachial, axillary and inguinal) lymph nodes, mesenteric lymph nodes, thymuses, and livers by meshing the organs through a 100 μm strainer (Corning, 07-201-432) with a syringe plunger. Cells in the colonic lamina propria were isolated as previously described^72^. Briefly, colons were cleaned by flushing the luminal content out with PBS using a syringe, defatted, opened up longitudinally and diced into 1–2 cm pieces. Tissues were then incubated in 25 mL IEL solution [1× PBS w/ 2% FBS (ThermoFisher, 35010CV), 10 mM HEPES buffer (ThermoFisher, MT 25-060-CI), 1% penicillin/streptomycin (ThermoFisher, MT 30-002-CI), 1% L-glutamine (ThermoFisher, MT 25-005-CI), plus 1 mM EDTA (Sigma-Aldrich, E4884) and 1 mM DTT (Sigma-Aldrich, D9779) added immediately before use] for 15 minutes at 37°C with vigorous shaking (250 rpm) to remove the epithelial fraction. Tissues were then retrieved, washed extensively, and digested in 25 mL LPL solution [1× RPMI 1640 w/2% FBS, 10 mM HEPES buffer, 1% penicillin/streptomycin, 1% L-glutamine, 0.2 U/mL collagenase A (Sigma, 11088793001) and 1 U/mL DNase I (Sigma-Aldrich, 10104159001)] for 30 minutes at 37°C with vigorous shaking (250 rpm). ¼inch ceramic beads (MP Biomedicals, 116540034) were added during this step (3–4 per sample) to facilitate tissue dissociation. The digested samples were passed through a 100 μm strainer, pelleted at 450 g for 5 minutes and washed extensively. Lungs were digested in the same fashion as the lamina propria fraction of the colons but for 45 minutes. Cells from non-lymphoid organs were centrifugated in 40% PBS-adjusted Percoll (v/v, ThermoFisher, 45-001-747) in PBS to enrich for lymphocytes. Erythrocytes in the spleen, lung, and liver samples were lysed with ACK lysis buffer [150 mM NH_4_Cl (Sigma-Aldrich, A9434), 10 mM KHCO_3_ (Sigma-Aldrich, P7682), 0.1 mM Na_2_EDTA, pH 7.4].

For fluorescence-activated cell sorting, cell suspension was made from pooled secondary lymphoid organs (spleen; peripheral and mesenteric lymph nodes) as above and CD4 T cells were enriched with the Dynabeads Flowcomp Mouse CD4 Kit (ThermoFisher, 11461D) according to manufacturer’s instructions, stained with antibodies, washed extensively, resuspended in isolation buffer (PBS w/ 2% FBS, 10 mM HEPES buffer, 1% L-glutamine, and 2 mM EDTA) containing 0.01% SYTOX Blue dead cell stain (ThermoFisher, S34857) to facilitate dead cell exclusion, and sorted on a FACSAria (BD) instrument. Treg cells (TCRβ^+^CD4^+^CD8^−^NK1.1^−^Foxp3^+^Thy1.1^−^), Treg “wannabes” (TCRβ^+^CD4^+^CD8^−^NK1.1^−^Thy1.1^+^Foxp3^−^) and naïve conventional CD4 T cells (TCRβ^+^CD4^+^CD8^−^NK1.1^−^Foxp3^−^Thy1.1^−^CD44^lo^CD62L^hi^) were sorted by gating on the respective populations.

### Histology and TUNEL assay

Sample embedding, sectioning, haematoxylin and eosin staining, pathology grading, and TUNEL (terminal deoxynucleotidyl transferase dUTP nick end labeling) assay were conducted at HistoWiz (Brooklyn, NY). Tissues were fixed in 10% neutral buffered formalin and embedded in paraffin before sectioned into 5-micron slices. Lymphocytic and acute (neutrophils) or chronic (monocytes) myeloid inflammation was blindly scored with the following criteria: 0-normal, 1-mild increase, 2-moderate increase, 3-medium increase, 4-sever increase.

For TUNEL assay, liver sample sections were processed under standardized conditions using the DeadEnd Fluorometric Detection System (Promega, G3250), and subsequent immunohistochemistry was carried out using BOND Polymer Refine Detection Kit (Leica, DS9800) according to manufacturers’ instructions. TUNEL^+^ cells were quantified with ImageJ v2.0.0-rc-69/1.52p.

### Measurement of serum albumin

Serum albumin level was measured by Laboratory of Comparative Pathology, SKI, using the Albumin kit (Beckman Coulter, OSR6102), according to manufacturer’s instruction.

### Flow cytometric analysis of cytokine production

To measure cytokine production after *ex vivo* restimulation, single cell suspensions were incubated at 37°C for 3–4 hours with 5% CO_2_ in 96-well flat-bottom plates in the presence of 50 ng/mL phorbol-12-myristate-13-acetate (PMA, Sigma-Aldrich, P8139) and 500 ng/mL ionomycin (Sigma-Aldrich, I0634) with 1 μg/mL brefeldin A (Sigma-Aldrich, B6542) and 2 μM monensin (Sigma-Aldrich, M5273) to inhibit ER and Golgi transport. Cells were then stained with Ghost Dye Violet 510 (Tonbo, 13–0870), Ghost Dye Red 780 (Tonbo, 13–0865), or Zombie NIR Flexible Viability Kit (Biolegend, 423106) in PBS for 10 minutes at 4°C to help identify dead cells and then with purified anti-Mouse CD16/CD32 (2.4G2, Tonbo, 70–0161) in staining buffer [0.5% (w/v) BSA, 2 mM EDTA, 10 mM HEPES, 0.02% NaN_3_ (Sigma-Aldrich, S2002) in 1× PBS] for 10 minutes at 4°C to block the Fc receptors. Samples were then incubated with fluorescently conjugated antibodies against cell surface antigens in staining buffer for 25 minutes at 4°C and then washed extensively. For accessing intracellular antigens, cells were fixed and permeabilized with the BD Cytofix/Cytoperm Kit for measuring cytokine production, or with the ThermoFisher Transcription Factor Fix/Perm Kit for staining cytosolic and nuclear antigens, according to manufacturers’ instructions. Samples were recorded on an LSR II cytometer (BD) using the BD FACS DIVA software v8.0 or an Aurora cytometer (Cytek) using the SpectroFlo software v2.2.0.3 and analyzed with FlowJo v10.6.1 (BD).

### *In vitro* suppression assay

A 2-fold titration series of FACS-sorted Treg cells starting from 40,000 cells/well was set up in U-bottom 96-well plates. 40,000 FACS-sorted, CellTrace Violet (ThermoFisher, C34571)-labeled naïve CD4 T cells and 100,000 erythrocyte-lysed splenocytes from *Tcrb*^*−/−*^*Tcrd*^*−/−*^ mice as antigen-presenting cells were then added to each well. α-mouse CD3 (145-2C11, BioXCell, BE0001-1) was then added to a final concentration of 1 μg/mL. Cells were incubated in a final volume of 200 μL complete RPMI w/ 10% FBS and with 5% CO_2_ at 37°C for 72 hours before analysis. Cells that have had more than 4 rounds of CTV dilution were considered divided for calculating Treg cell-mediated suppression using the following formula:

$$\%Suppression\;(Sample\;X)=\frac{\%divided\;\left(no\;Treg\right)-\%divided\;(Sample\;X)}{\%divided\;\left(no\;Treg\right)}$$

### Bulk RNA sequencing and data analysis

Doubly sorted cells were directly placed into TRIzol reagent (ThermoFisher, 15596–018) for subsequent RNA extraction. RNA was precipitated with isopropanol and linear acrylamide, washed with 75% ethanol, and resuspended in RNase-free water. After RiboGreen quantification and quality control by Agilent BioAnalyzer, 2ng of total RNA underwent amplification using the SMART-Seq v4 Ultra Low Input RNA Kit (Clontech, 63488), with 12 cycles of amplification. 3.8 – 4 ng of amplified cDNA was then used to prepare libraries with the KAPA Hyper Prep Kit (Kapa Biosystems, KK8504) using 8 cycles of PCR. Barcoded samples were run on a HiSeq 4000 instrument in a 50bp/50bp paired-end run, using the HiSeq 3000/4000 SBS Kit (Illumina). An average of 41 million paired reads were generated per sample with % mRNA bases per sample ranging from 67% to 77%. In experiments shown in Fig. 3, Treg cells (TCRβ^+^CD4^+^Foxp3^+^) from *Foxp3*^DTR-GFP/y^ and *Foxp3*^LSL/y^*Cd4*^creERT2*/+*^ mice and Treg “wannabe” (TCRβ^+^CD4^+^Foxp3^−^Thy1.1^+^) cells from *Foxp3*^LSL/y^*Cd4*^*+/+*^ mice were analyzed (2–3 biological replicates). In experiments shown in Extended Data Fig. 6, Treg “wannabes” from 3 *Foxp3*^LSL*/+*^ mice and 2 *Foxp3*^LSL*/*DTR-GFP^ mice were analyzed. RNA-seq reads from fastq files were aligned to the reference mouse genome GRCm38 (https://www.ncbi.nlm.nih.gov/assembly/GCF_000001635.20/) using the STAR aligner v2.7.3a^73^, and local realignment was performed using the Genome Analysis Toolkit v4.1.4.1^74^. For each sample, raw count of reads per gene was measured using R v4.0.2, and the DESeq2 R package v1.28.1^75^ was used to perform differential gene expression analysis across different conditions. A cutoff of 0.05 was set on the obtained FDR-adjusted p-values to get the significant genes of each comparison. All detectable genes were rlog-normalized and then used for the principal component analysis.

### Single cell RNA sequencing and data analysis

#### Library Preparation and sequencing

Library preparation and sequencing for the scRNA-Seq of doubly-FACS-sorted Treg cells isolated from the secondary lymphoid organs of 7 months post-4-OHT treatment *Foxp3*^DTR-GFP^ (cells pooled from 5 mice) and *Foxp3*^LSL^*Cd4*^creERT2*/+*^ mice (cells pooled from 4 mice) were performed by the Single Cell Research Initiative, SKI, using 10× genomics Chromium Single Cell 3’ Library & Gel bead Kit V3 according to manufacturer’s protocol. Cells, with a mean viability of 75%, were encapsulated in microfluidic droplets at a dilution of ~60 cells/μL with the multiplet fraction being 3.5%. After the RT step, the barcoded-cDNA was purified with DynaBeads, followed by 12-cycles of PCR-amplification (98°C for 180 s; [98°C for 15 s, 67°C for 20 s, 72°C for 60 s] x 12-cycles; 72°C for 60 s). Next, 50 ng of PCR-amplified barcoded cDNA was fragmented with the reagents provided in the kit and purified with SPRI beads to obtain an average fragment size of 600 bp. The fragmented DNA was ligated to sequencing adapters which was then indexed with PCR (98°C for 45 s; [98°C for 20 s, 54°C for 30 s, 72°C for 20 s] x 10 cycles; 72°C for 60 s). The resulting DNA library was double-size purified (0.6–0.8X) with SPRI beads and sequenced on an Illumina NovaSeq 6000 System (R1 – 26 cycles, i7 – 8 cycles, R2 – 96 cycles) at a depth of 210 million reads per sample (average reads per single cell being 31,000 and average reads per transcript 3.96 – 4.10), with a median sequencing saturation of 74%.

#### Data Pre-processing

Fastq files were processed using Cell Ranger v3.0 (10x Genomics) and reads were aligned to the mouse genome mm10 from ENSEMBL GRCm38 that was modified to include sequences corresponding to the coding region and the 3’UTR of the *R26*^Tom^ allele. Cells containing over 5% mitochondria-derived transcripts were filtered out, resulting in 3,634 *Foxp3*^DTR-GFP^ cells and 3,390 *Foxp3*^LSL^ cells that passed QC metrics, with a median of 3,580 molecules/cell. Cells with total molecule counts below 1000, as determined by the lower mode of the distribution of total molecules per cell, were additionally filtered out to remove putative empty droplets. Genes that were expressed in more than 10 cells were retained for further analysis. The resulting count matrices from both samples were then combined, resulting in a final set of 7,024 cells x 12,432 genes, and normalized to median library size, where library size is defined as total molecule counts per cell. The normalized data are then log transformed as log (counts + 1) for downstream analysis.

#### Principal component analysis

For dimensionality reduction and visualization of data, we further excluded genes with very low or very high expression of transcripts (log average expression <0.02 or >3 and dispersion >0.15), and principal component analysis was then performed on the log-transformed normalized data. Using 40 principal components, where the number of principal components was determined by the knee-point in eigenvalues, yielded a good representation of the variability in the data.

#### MAGIC imputation

To account for missing values in scRNA-seq due to a high dropout rate, we employed MAGIC v0.1.1, a method of “de-noising” and imputing missing expression values through data diffusion between cells with similar covariate gene relationships^76^. We constructed the diffusion map matrix using k = 30, ka = 10, and t = 6 as input parameters, where t specifies the number of times the affinity matrix is powered for diffusion.

#### Diffusion components and pseudotime calculation

Instead of constructing a tSNE map using 40 PCs, we followed the strategy outlined by Setty et al.^41^ In order to characterize potential pseudotime non-linear trajectories and to visualize single-cell gene expression in a UMAP embedding of diffusion components. Based on the eigen gap, we chose to use 15 diffusion components for downstream analysis in Palantir v1.0.0 and for calculating diffusion distances. We scaled each included diffusion component by the factor λ/(1-λ) where λ is the associated eigenvalue, to reflect ‘multi-scale’ diffusion distances. Then, we calculate each cell position in pseudotime based on modeling cell fate in a continuous probabilistic model.

#### Clustering and gene ranking

Clustering of cells was performed using PhenoGraph v1.5.7^77^ setting k = 15 nearest neighbors. A cluster was removed because of its disparity with the rest of the data (t-SNE projected this cluster as a separate component that comprised cells from both populations), and those cells also had a relatively lower number of total molecules compared with other populations. Significant differentially expressed genes in each cluster were identified using *t*-test (where the variance of small groups is overestimated), which was implemented in Single-Cell Analysis in Python (Scanpy) v1.7.2^78^ with default parameters.

#### tdTomato expression and gene set module score calculation

Because of a high dropout rate of single cell sequencing, we performed MAGIC imputation of tdTomato expression (as described above) only for *Foxp3*^DTR-GFP^ cells since the overwhelming majority of the *Foxp3*^LSL^ cells were tdTomato^+^ which could potentially cause over-imputation. A cutoff of 1.04 was set for the imputed tdTomato expression where any cells with higher expression were categorized as tdTomato^+^(~15% of cells) and those with lower expression as tdTomato^−^ (~85% of cells) in agreement with flow cytometric measurements. Gene set module scores for Il2-Stat5 (GSEA HALLMARK_IL2_STAT5_SIGNALING) and Wnt/β-catenin (GSEA HALLMARK_WNT_BETA_CATENIN_SIGNALING) were calculated with the AddModuleScore function in Seurat v 3.1.5^79^ using the default parameters.

### TCR sequencing and data analysis

Bulk sequencing of the TCRα chain was performed using a 5’ RACE-based method as previously described^36^. Briefly, 70,000 rescued Treg cells (TCRβ^+^CD4^+^GFP^+^Thy1.1^−^) from *Foxp3*^LSL/y^*Cd4*^creERT2^ mice were FACS-sorted from pooled spleen and lymph nodes at different time points post 4-OHT administration. Total RNA was extracted using the RNAeasy Plus Micro Kit (Qiagen 74034) according to the manufacturer’s instructions. cDNA was synthesized using the SMARTScribe Reverse Transcriptase (Clontech 639537) with a mixture of oligo(dT)_24_ and primers targeting the mouse TCRα constant regions. A template switching DNA-RNA hybrid oligo containing 12 random nucleotides was used to hybridize onto the 3’ end of the first stand cDNA and to barcode the individual cDNA molecules. After removal of the hybrid oligo with Uracil-DNA glycosylase (New England Biolabs M0280), the cDNA was further PCR amplified to introduce sample barcodes, sequencing primer binding sites, as well as the Illumina P5 and P7 sequencing adaptors. The final PCR products were separated on a 1% agarose gel and a single band around 700 bp was cut and purified using the NucleoSpin Gel and PCR Clean-up Kit (Clontech 740609). After PicoGreen quantification and quality control by Agilent TapeStation, libraries were pooled in equimolar ratios and run on a MiSeq in a PE200 run, using the MiSeq Reagent Kit v3 (600 Cycles) (Illumina). The loading concentration was 7–18 pM and a 10–20% spike-in of PhiX was added to the run to increase diversity and for quality control purposes (libraries were sequenced 3 times; loading concentration and PhiX spike-in amounts were adjusted based on initial performance). The runs yielded an average of 815K reads per sample.

The fastq files containing the TCR sequencing reads were aligned using the MiXCR software v3.0.13 to reference the V, D and J genes of mouse T cell receptors^80^. MiXCR was also used to assemble clonotypes using alignments obtained from the previous step in order to extract the highly variable CDR3 region. The resulting clonotypes were pre-processed using VDJtools 1.2.1^81^ in two steps. The first is a frequency-based correction to eliminate erroneous clonotypes. The algorithm searched the sample for clonotype pairs that differ by up to 2 mismatches. In case the ratio of the smallest to the largest clonotype sizes was lower than a specified threshold of 0.05^(number of mismatches), correction was performed by discarding the smaller clonotypes. The second step was to filter non-functional clonotypes. Specifically, the ones that contained a stop codon or frameshift in their receptor sequences were discarded. The resulting clonotypes and clone sizes were used to calculate the Simpson index and Gini coefficient. Two different clonotype matching strategies were used: 1) V, J and CDR3 nucleotide sequences and 2) the CDR3 amino-acid sequence.

### Statistics

Statistical significance was determined using tests indicated in the respective figure legends. P-values for *t*-test and Mantel-Cox test on flow cytometric and survival data were calculated with GraphPad Prism 7 and had been corrected for multiple hypothesis testing using the Holm-Sidak method, when applicable. P-values for ANOVA were computed with R for Fig. 2b and GraphPad Prism 7 elsewhere and had been corrected for multiple hypothesis testing using the Tukey method (when using R and Prism), the Dunnett’s method or the Sidak method (when using Prism, according to its recommendations). P-values for Kolmogorov-Smirnov test were calculated with R. Throughout the entire study, error bars represent mean ± s.e.m., and the following notation was used to report statistical significance: ns, non-significant; *, p < 0.05; **, p < 0.01; ***, p < 0.001; ****, p < 0.0001.

### Data availability

All sequencing data generated in this study can be accessed at GEO under accession number GSE179710. The custom mouse genome used for scRNA-seq analysis, which was generated by adding the tdTomato sequence to GRCm38 (https://www.ncbi.nlm.nih.gov/assembly/GCF_000001635.20/), is also available at GEO. The following GSEA gene sets were used for data analysis: GO_CELL_CYCLE_G1_S_PHASE_TRANSITION, GO_CELL_CYCLE_G2_M_PHASE_TRANSITION, GO_DNA_REPLICATION, HALLMARK_APOPTOSIS, HALLMARK_FATTY_ACID_METABOLISM, HALLMARK_G2M_CHECKPOINT, HALLMARK_GLYCOLYSIS, HALLMARK_IL2_STAT5_SIGNALING, HALLMARK_OXIDATIVE_PHOSPHORYLATION, HALLMARK_PI3K_AKT_MTOR_SIGNALING, HALLMARK_REACTIVE_OXYGEN_SPECIES_PATHWAY, HALLMARK_TGF_BETA_SIGNALING, HALLMARK_WNT_BETA_CATENIN_SIGNALING, KEGG_CITRATE_CYCLE_TCA_CYCLE, KEGG_PURINE_METABOLISM, REACTOME_EUKARYOTIC_TRANSLATION_INITIATION.

### Code availability

All custom scripts are available upon request to the corresponding authors.

## Extended Data

## Supplementary Material

Supplementary Table 2

Supplementary Table 1

Supplementary Figure 1
