# Supplementary material for: Regulatory T Cells Function in Established Systemic Inflammation and Reverse Fatal Autoimmunity
Source: Nat Immunol. Author manuscript; Available in PMC 2022 Aug 1. (PMC9341271; doi:10.1038/s41590-021-01001-4)
Supplement: Supplementary Table 2 [file NIHMS1820168-supplement-Supplementary_Table_2.pdf]

Figure	Panel	Sample size
Fig. 1	Fig. 1d	Week 0, $Foxp3^{LSL/y}Cd4^{wt}$, n=6; $Foxp3^{DTR\text{-}GFP/y}Cd4^{creERT2}$, n=7; $Foxp3^{LSL/y}Cd4^{creERT2}$, n=7.<br>Week 1, $Foxp3^{LSL/y}Cd4^{wt}$, n=6; $Foxp3^{DTR\text{-}GFP/y}Cd4^{creERT2}$, n=8; $Foxp3^{LSL/y}Cd4^{creERT2}$, n=8.<br>Week 2, $Foxp3^{LSL/y}Cd4^{wt}$, n=6; $Foxp3^{DTR\text{-}GFP/y}Cd4^{creERT2}$, n=12; $Foxp3^{LSL/y}Cd4^{creERT2}$, n=8.<br>Week 4, $Foxp3^{LSL/y}Cd4^{wt}$, n=6; $Foxp3^{DTR\text{-}GFP/y}Cd4^{creERT2}$, n=3; $Foxp3^{LSL/y}Cd4^{creERT2}$, n=5.
	Fig. 1e	Week 0, $Foxp3^{LSL/y}Cd4^{wt}$, n=6; $Foxp3^{DTR\text{-}GFP/y}Cd4^{creERT2}$, n=5; $Foxp3^{LSL/y}Cd4^{creERT2}$, n=5.<br>Week 1, $Foxp3^{LSL/y}Cd4^{wt}$, n=6; $Foxp3^{DTR\text{-}GFP/y}Cd4^{creERT2}$, n=8; $Foxp3^{LSL/y}Cd4^{creERT2}$, n=8.<br>Week 2, $Foxp3^{LSL/y}Cd4^{wt}$, n=5; $Foxp3^{DTR\text{-}GFP/y}Cd4^{creERT2}$, n=11; $Foxp3^{LSL/y}Cd4^{creERT2}$, n=8.<br>Week 4, $Foxp3^{LSL/y}Cd4^{wt}$, n=6; $Foxp3^{DTR\text{-}GFP/y}Cd4^{creERT2}$, n=6; $Foxp3^{LSL/y}Cd4^{creERT2}$, n=7.
	Fig. 1f	Week 0, $Foxp3^{LSL/y}Cd4^{wt}$, n=8; $Foxp3^{DTR\text{-}GFP/y}Cd4^{creERT2}$, n=4; $Foxp3^{LSL/y}Cd4^{creERT2}$, n=6.<br>Week 1, $Foxp3^{LSL/y}Cd4^{wt}$, n=7; $Foxp3^{DTR\text{-}GFP/y}Cd4^{creERT2}$, n=2; $Foxp3^{LSL/y}Cd4^{creERT2}$, n=3.<br>Week 2, $Foxp3^{LSL/y}Cd4^{wt}$, n=4; $Foxp3^{DTR\text{-}GFP/y}Cd4^{creERT2}$, n=6; $Foxp3^{LSL/y}Cd4^{creERT2}$, n=8.<br>Week 4, $Foxp3^{LSL/y}Cd4^{wt}$, n=6; $Foxp3^{DTR\text{-}GFP/y}Cd4^{creERT2}$, n=6; $Foxp3^{LSL/y}Cd4^{creERT2}$, n=7.
	Fig. 1g	$Foxp3^{LSL/y}Cd4^{wt}$, n=7; $Foxp3^{DTR\text{-}GFP/y}Cd4^{creERT2}$, n=6; $Foxp3^{LSL/y}Cd4^{creERT2}$, n=7.
	Fig. 1i	$Foxp3^{LSL/y}Cd4^{wt}$, n=4; $Foxp3^{DTR\text{-}GFP/y}Cd4^{creERT2}$, n=2; $Foxp3^{LSL/y}Cd4^{creERT2}$, n=4.
Fig. 2	Fig. 2b	Week 0, $Foxp3^{DTR\text{-}GFP/LSL}Cd4^{wt}$, n=6; $Foxp3^{DTR\text{-}GFP/WT}Cd4^{creERT2}$, n=4; $Foxp3^{DTR\text{-}GFP/LSL}Cd4^{creERT2}$, n=3.<br>Week 1, $Foxp3^{DTR\text{-}GFP/LSL}Cd4^{wt}$, n=5; $Foxp3^{DTR\text{-}GFP/WT}Cd4^{creERT2}$, n=4; $Foxp3^{DTR\text{-}GFP/LSL}Cd4^{creERT2}$, n=4.<br>Week 2, $Foxp3^{DTR\text{-}GFP/WT}Cd4^{creERT2}$, n=4; $Foxp3^{DTR\text{-}GFP/LSL}Cd4^{creERT2}$, n=3.<br>Week 5, $Foxp3^{DTR\text{-}GFP/LSL}Cd4^{wt}$, n=5; $Foxp3^{DTR\text{-}GFP/WT}Cd4^{creERT2}$, n=4; $Foxp3^{DTR\text{-}GFP/LSL}Cd4^{creERT2}$, n=5.
	Fig. 2c	Week 1, $Foxp3^{DTR\text{-}GFP/LSL}Cd4^{wt}$, n=4; $Foxp3^{DTR\text{-}GFP/WT}Cd4^{creERT2}$, n=5; $Foxp3^{DTR\text{-}GFP/LSL}Cd4^{creERT2}$, n=4.<br>Week 5, $Foxp3^{DTR\text{-}GFP/WT}Cd4^{creERT2}$, n=5; $Foxp3^{DTR\text{-}GFP/LSL}Cd4^{creERT2}$, n=5.
	Fig. 2d	Week 0, $Foxp3^{DTR\text{-}GFP/LSL}Cd4^{wt}$, n=6; $Foxp3^{DTR\text{-}GFP/WT}Cd4^{creERT2}$, n=4; $Foxp3^{DTR\text{-}GFP/LSL}Cd4^{creERT2}$, n=4.<br>Week 5, $Foxp3^{DTR\text{-}GFP/WT}Cd4^{creERT2}$, n=5; $Foxp3^{DTR\text{-}GFP/LSL}Cd4^{creERT2}$, n=5.
	Fig. 2e	Week 0, $Foxp3^{DTR\text{-}GFP/LSL}Cd4^{wt}$, n=6; $Foxp3^{DTR\text{-}GFP/WT}Cd4^{creERT2}$, n=4; $Foxp3^{DTR\text{-}GFP/LSL}Cd4^{creERT2}$, n=4.<br>Week 5, $Foxp3^{DTR\text{-}GFP/WT}Cd4^{creERT2}$, n=5; $Foxp3^{DTR\text{-}GFP/LSL}Cd4^{creERT2}$, n=5.
Fig. 3	Fig. 3a	Week 1, $Foxp3^{DTR\text{-}GFP/y}Cd4^{creERT2}$, n=8; $Foxp3^{LSL/y}Cd4^{creERT2}$, n=6.<br>Week 2, $Foxp3^{DTR\text{-}GFP/y}Cd4^{creERT2}$, n=11; $Foxp3^{LSL/y}Cd4^{creERT2}$, n=8.<br>Week 4, $Foxp3^{DTR\text{-}GFP/y}Cd4^{creERT2}$, n=3; $Foxp3^{LSL/y}Cd4^{creERT2}$, n=5.

	Fig. 3f	$Foxp3^{LSL/WT}Cd4^{creERT2}$ GFP+, n=3; $Foxp3^{LSL/WT}Cd4^{creERT2}$ GFP-, n=3;<br>$Foxp3^{DTR/WT}Cd4^{creERT2}$ GFP+, n=3; $Foxp3^{DTR/WT}Cd4^{creERT2}$ GFP-, n=3.
	Fig. 3h	Week 1, $Foxp3^{DTR/WT}Cd4^{creERT2}$, n=5; $Foxp3^{DTR/LSL}Cd4^{creERT2}$, n=4.<br>Week 2, $Foxp3^{DTR/WT}Cd4^{creERT2}$, n=3; $Foxp3^{DTR/LSL}Cd4^{creERT2}$, n=4.<br>Week 5, $Foxp3^{DTR/WT}Cd4^{creERT2}$, n=8; $Foxp3^{DTR/LSL}Cd4^{creERT2}$, n=9.
	Fig. 3j	$Foxp3^{DTR/WT}Cd4^{creERT2}$, n=5; $Foxp3^{DTR/LSL}Cd4^{creERT2}$, n=4.
Fig. 4	Fig. 4b	$Foxp3^{DTR/y}Cd4^{creER/+}$, n=5; $Foxp3^{LSL/y}Cd4^{creER/+}$, n=5.
	Fig. 4c	Treg percentage, $Foxp3^{DTR/y}Cd4^{creER/+}$, n=9; $Foxp3^{LSL/y}Cd4^{creER/+}$, n=7.<br>Treg cell number, $Foxp3^{DTR/y}Cd4^{creER/+}$, n=5; $Foxp3^{LSL/y}Cd4^{creER/+}$, n=5.
	Fig. 4d	$Foxp3^{DTR/y}Cd4^{creER}$ Tconv, n=6; $Foxp3^{LSL/y}Cd4^{creER}$ Tconv, n=7;<br>$Foxp3^{DTR/y}Cd4^{creER}$ Treg, n=4; $Foxp3^{LSL/y}Cd4^{creERT2}$ Treg, n=7;<br>$Foxp3^{LSL/y}Cd4^{creER}$ Wannabe, n=7.
	Fig. 4f	$Foxp3^{DTR/y}Cd4^{creER}$, n=9; $Foxp3^{LSL/y}Cd4^{creER}$ Tconv, n=7.
	Fig. 4g	$Foxp3^{DTR/y}Cd4^{creER}$, n=9; $Foxp3^{LSL/y}Cd4^{creER}$ Tconv, n=7.
	Fig. 4h	$Foxp3^{DTR/y}Cd4^{creER}$, n=5; $Foxp3^{LSL/y}Cd4^{creER}$ Tconv, n=5.
Fig. 5	Fig. 5c	7 Days, $Foxp3^{DTR/y}Cd4^{creER}$, n=9; $Foxp3^{LSL/y}Cd4^{creER}$ Tconv, n=9.<br>1 Mon, $Foxp3^{DTR/y}Cd4^{creER}$, n=4; $Foxp3^{LSL/y}Cd4^{creER}$ Tconv, n=9.<br>4 Mon, $Foxp3^{DTR/y}Cd4^{creER}$, n=4; $Foxp3^{LSL/y}Cd4^{creER}$ Tconv, n=5.<br>7 Mon, $Foxp3^{DTR/y}Cd4^{creER}$, n=4; $Foxp3^{LSL/y}Cd4^{creER}$ Tconv, n=5.
	Fig. 5j	$Foxp3^{LSL/y}$, 3390 cells; $Foxp3^{DTR/y}$ $tdTomato^-$, 2910 cells; $Foxp3^{DTR/y}$<br>$tdTomato^+$, 724 cells.
Fig. 6	Fig. 6f	$Foxp3^{DTR/y}ROSA^{Tom}Cd4^{creERT2}$, n=5; $Foxp3^{LSL/y}ROSA^{Tom}Cd4^{creERT2}$, n=2.
	Fig. 6g	$γREG^+$ Treg percentage, n=5.<br>$γREG^+$ Treg cell number, n=5.
	Fig. 6h	$Foxp3^{DTR\text{-}GFP}Cd4^{creERT2}R26^{Tom}$, adult, n=5; perinatal, n=5.<br>$Foxp3^{creERT2}R26^{Tom}$, adult, n=3; perinatal, n=6.
Extended Data Fig. 1	Extended Data Fig. 1f	n=5.
	Extended Data Fig. 1g	No Treg, n=3; $Thy1.1^+$ ($Foxp3^{LSL/y}Cd4^{creERT2/+}$), n=4; $Thy1.1^+$<br>($Foxp3^{LSL/y}Cd4^{+/+}$), n=4; Treg ($Foxp3^{GFP}$), n=4.
Extended Data Fig. 2	Extended Data Fig. 2a	n=6.
	Extended Data Fig. 2d	Week 0, $Foxp3^{LSL/y}Cd4^{wt}$, n=6; $Foxp3^{DTR\text{-}GFP/y}Cd4^{creERT2}$, n=7;<br>$Foxp3^{LSL/y}Cd4^{creERT2}$, n=7.<br>Week 1, $Foxp3^{LSL/y}Cd4^{wt}$, n=6; $Foxp3^{DTR\text{-}GFP/y}Cd4^{creERT2}$, n=8;<br>$Foxp3^{LSL/y}Cd4^{creERT2}$, n=8.<br>Week 2, $Foxp3^{LSL/y}Cd4^{wt}$, n=6; $Foxp3^{DTR\text{-}GFP/y}Cd4^{creERT2}$, n=12;<br>$Foxp3^{LSL/y}Cd4^{creERT2}$, n=8.<br>Week 4, $Foxp3^{LSL/y}Cd4^{wt}$, n=6; $Foxp3^{DTR\text{-}GFP/y}Cd4^{creERT2}$, n=3;<br>$Foxp3^{LSL/y}Cd4^{creERT2}$, n=5.
	Extended Data Fig. 2e	Week 0, $Foxp3^{LSL/y}Cd4^{wt}$, n=6; $Foxp3^{DTR\text{-}GFP/y}Cd4^{creERT2}$, n=7;<br>$Foxp3^{LSL/y}Cd4^{creERT2}$, n=7.<br>Week 1, $Foxp3^{LSL/y}Cd4^{wt}$, n=6; $Foxp3^{DTR\text{-}GFP/y}Cd4^{creERT2}$, n=8;<br>$Foxp3^{LSL/y}Cd4^{creERT2}$, n=8.<br>Week 2, $Foxp3^{LSL/y}Cd4^{wt}$, n=6; $Foxp3^{DTR\text{-}GFP/y}Cd4^{creERT2}$, n=12;<br>$Foxp3^{LSL/y}Cd4^{creERT2}$, n=8.

		Week 4, *Foxp3*$^{\text{LSL/y}}$*Cd4*^{wt}, n=6; *Foxp3*$^{\text{DTR-GFP/y}}$*Cd4*$^{\text{creERT2}}$, n=3; *Foxp3*$^{\text{LSL/y}}$*Cd4*$^{\text{creERT2}}$, n=7.
	Extended Data Fig. 2f	Week 0, *Foxp3*$^{\text{LSL/y}}$*Cd4*^{wt}, n=6; *Foxp3*$^{\text{DTR-GFP/y}}$*Cd4*$^{\text{creERT2}}$, n=5; *Foxp3*$^{\text{LSL/y}}$*Cd4*$^{\text{creERT2}}$, n=5.<br>Week 1, *Foxp3*$^{\text{LSL/y}}$*Cd4*^{wt}, n=6; *Foxp3*$^{\text{DTR-GFP/y}}$*Cd4*$^{\text{creERT2}}$, n=8; *Foxp3*$^{\text{LSL/y}}$*Cd4*$^{\text{creERT2}}$, n=8.<br>Week 2, *Foxp3*$^{\text{LSL/y}}$*Cd4*^{wt}, n=5; *Foxp3*$^{\text{DTR-GFP/y}}$*Cd4*$^{\text{creERT2}}$, n=11; *Foxp3*$^{\text{LSL/y}}$*Cd4*$^{\text{creERT2}}$, n=8.<br>Week 4, *Foxp3*$^{\text{LSL/y}}$*Cd4*^{wt}, n=6; *Foxp3*$^{\text{DTR-GFP/y}}$*Cd4*$^{\text{creERT2}}$, n=6; *Foxp3*$^{\text{LSL/y}}$*Cd4*$^{\text{creERT2}}$, n=7.
	Extended Data Fig. 2g	*Foxp3*$^{\text{LSL/y}}$*Cd4*^{wt}, n=7; *Foxp3*$^{\text{DTR-GFP/y}}$*Cd4*$^{\text{creERT2}}$, n=6; *Foxp3*$^{\text{LSL/y}}$*Cd4*$^{\text{creERT2}}$, n=7.
Extended Data Fig. 3	Extended Data Fig. 3b	*Treg number, Foxp3*$^{\text{DTR-GFP/y}}$*Cd4*$^{\text{creERT2}}$ control, n=8; *Foxp3*$^{\text{DTR-GFP/y}}$*Cd4*$^{\text{creERT2}}$ FTY720, n=12; *Foxp3*$^{\text{LSL/y}}$*Cd4*$^{\text{creERT2}}$ control, n=9; *Foxp3*$^{\text{LSL/y}}$*Cd4*$^{\text{creERT2}}$ FTY720, n=5.<br>tdTomato+ percentage, *Foxp3*$^{\text{DTR-GFP/y}}$*Cd4*$^{\text{creERT2}}$ control, n=2; *Foxp3*$^{\text{DTR-GFP/y}}$*Cd4*$^{\text{creERT2}}$ FTY720, n=10; *Foxp3*$^{\text{LSL/y}}$*Cd4*$^{\text{creERT2}}$ control, n=9; *Foxp3*$^{\text{LSL/y}}$*Cd4*$^{\text{creERT2}}$ FTY720, n=5.
	Extended Data Fig. 3c	*Foxp3*$^{\text{LSL/y}}$*Cd4*^{wt}, n=6; *Foxp3*$^{\text{DTR-GFP/y}}$*Cd4*$^{\text{creERT2}}$, n=12; *Foxp3*$^{\text{LSL/y}}$*Cd4*$^{\text{creERT2}}$, n=5.
	Extended Data Fig. 3d	*Foxp3*$^{\text{LSL/y}}$*Cd4*^{wt}, n=6; *Foxp3*$^{\text{DTR-GFP/y}}$*Cd4*$^{\text{creERT2}}$, n=12; *Foxp3*$^{\text{LSL/y}}$*Cd4*$^{\text{creERT2}}$, n=5.
Extended Data Fig. 4	Extended Data Fig. 4d	Week 0, *Foxp3*$^{\text{LSL/y}}$*Cd4*^{wt}, n=8; *Foxp3*$^{\text{DTR-GFP/y}}$*Cd4*$^{\text{creERT2}}$, n=8; *Foxp3*$^{\text{LSL/y}}$*Cd4*$^{\text{creERT2}}$, n=5.<br>Week 4, *Foxp3*$^{\text{LSL/y}}$*Cd4*^{wt}, n=3; *Foxp3*$^{\text{DTR-GFP/y}}$*Cd4*$^{\text{creERT2}}$, n=6; *Foxp3*$^{\text{LSL/y}}$*Cd4*$^{\text{creERT2}}$, n=4.
	Extended Data Fig. 4e	Week 0, *Foxp3*$^{\text{LSL/y}}$*Cd4*^{wt}, n=11; *Foxp3*$^{\text{DTR-GFP/y}}$*Cd4*$^{\text{creERT2}}$, n=10; *Foxp3*$^{\text{LSL/y}}$*Cd4*$^{\text{creERT2}}$, n=8.<br>Week 4, *Foxp3*$^{\text{LSL/y}}$*Cd4*^{wt}, n=4; *Foxp3*$^{\text{DTR-GFP/y}}$*Cd4*$^{\text{creERT2}}$, n=6; *Foxp3*$^{\text{LSL/y}}$*Cd4*$^{\text{creERT2}}$, n=5.
Extended Data Fig. 5	Extended Data Fig. 5c	Spleen, *Foxp3*^{WT} *Treg*, n=8; *Foxp3*^{LSL} Treg, n=9; Wannabe, n=9.<br>pLN, *Foxp3*^{WT} *Treg*, n=8; *Foxp3*^{LSL} Treg, n=9; Wannabe, n=9.<br>mLN, *Foxp3*^{WT} *Treg*, n=7; *Foxp3*^{LSL} Treg, n=9; Wannabe, n=9.<br>Lung, *Foxp3*^{WT} *Treg*, n=3; *Foxp3*^{LSL} Treg, n=4; Wannabe, n=9.<br>Liver, *Foxp3*^{WT} *Treg*, n=3; *Foxp3*^{LSL} Treg, n=4; Wannabe, n=4.<br>Colon LP, *Foxp3*^{WT} *Treg*, n=3; *Foxp3*^{LSL} Treg, n=4; Wannabe, n=4.
Extended Data Fig. 7	Extended Data Fig. 7a	*Foxp3*$^{\text{DTR-GFP/y}}$*Cd4*$^{\text{creERT2}}$, n=10; *Foxp3*$^{\text{LSL/y}}$*Cd4*$^{\text{creERT2}}$, n=10.
	Extended Data Fig. 7c	*Foxp3*$^{\text{DTR-GFP/y}}$*Cd4*$^{\text{creERT2}}$, n=2; *Foxp3*$^{\text{LSL/y}}$*Cd4*$^{\text{creERT2}}$, n=2.
	Extended Data Fig. 7d	*Foxp3*$^{\text{DTR-GFP/y}}$*Cd4*$^{\text{creERT2}}$, n=5; *Foxp3*$^{\text{LSL/y}}$*Cd4*$^{\text{creERT2}}$, n=5.
	Extended Data Fig. 7e	*Foxp3*$^{\text{DTR-GFP/y}}$*Cd4*$^{\text{creERT2}}$, n=5; *Foxp3*$^{\text{LSL/y}}$*Cd4*$^{\text{creERT2}}$, n=5.
	Extended Data Fig. 7f	*Foxp3*$^{\text{DTR-GFP/y}}$*Cd4*$^{\text{creERT2}}$, n=5; *Foxp3*$^{\text{LSL/y}}$*Cd4*$^{\text{creERT2}}$, n=5.

Extended Data Fig. 8	Extended Data Fig. 8a	Spleen, pLN, lung, liver, colon LP, *Foxp3*$^{DTR\text{-}GFP/y}$*Cd4*^{creERT2}, n=5; *Foxp3*$^{LSL/y}$*Cd4*^{creERT2}, n=5.<br>mLN, *Foxp3*$^{DTR\text{-}GFP/y}$*Cd4*^{creERT2}, n=4; *Foxp3*$^{LSL/y}$*Cd4*^{creERT2}, n=5.
	Extended Data Fig. 8b	Spleen, pLN, lung, liver, colon LP, *Foxp3*$^{DTR\text{-}GFP/y}$*Cd4*^{creERT2}, n=5; *Foxp3*$^{LSL/y}$*Cd4*^{creERT2}, n=5.<br>mLN, *Foxp3*$^{DTR\text{-}GFP/y}$*Cd4*^{creERT2}, n=4; *Foxp3*$^{LSL/y}$*Cd4*^{creERT2}, n=5.
	Extended Data Fig. 8c	Spleen, pLN, lung, liver, colon LP, *Foxp3*$^{DTR\text{-}GFP/y}$*Cd4*^{creERT2}, n=5; *Foxp3*$^{LSL/y}$*Cd4*^{creERT2}, n=5.<br>mLN, *Foxp3*$^{DTR\text{-}GFP/y}$*Cd4*^{creERT2}, n=4; *Foxp3*$^{LSL/y}$*Cd4*^{creERT2}, n=5.
	Extended Data Fig. 8d	*Foxp3*$^{DTR\text{-}GFP/y}$*Cd4*^{creERT2}, n=5; *Foxp3*$^{LSL/y}$*Cd4*^{creERT2}, n=5.
	Extended Data Fig. 8f	1.5 mon, n=2; 3 mon, n=4, 5 mon, n=2; 7 mon, n=5.
	Extended Data Fig. 8g	1.5 mon, n=2; 3 mon, n=4, 5 mon, n=2; 7 mon, n=5.
Extended Data Fig. 9	Extended Data Fig. 9b	*Foxp3*$^{DTR\text{-}GFP/y}$*Cd4*^{creERT2}, n=5; *Foxp3*$^{LSL/y}$*Cd4*^{creERT2}, n=5.
Extended Data Fig. 10	Extended Data Fig. 10c	Spleen, pLN, mLN, n=4.
	Extended Data Fig. 10e	Spleen, pLN, mLN, n=4.
	Extended Data Fig. 10f	Spleen, pLN, mLN, lung, liver, colon LP, thymus, n=5.
	Extended Data Fig. 10g	Perinate, n=4; adult, n=4.
