# Supplementary figures and images for: Regulatory T Cells Function in Established Systemic Inflammation and Reverse Fatal Autoimmunity
Source: Nat Immunol. Author manuscript; Available in PMC 2022 Aug 1. (PMC9341271; doi:10.1038/s41590-021-01001-4)

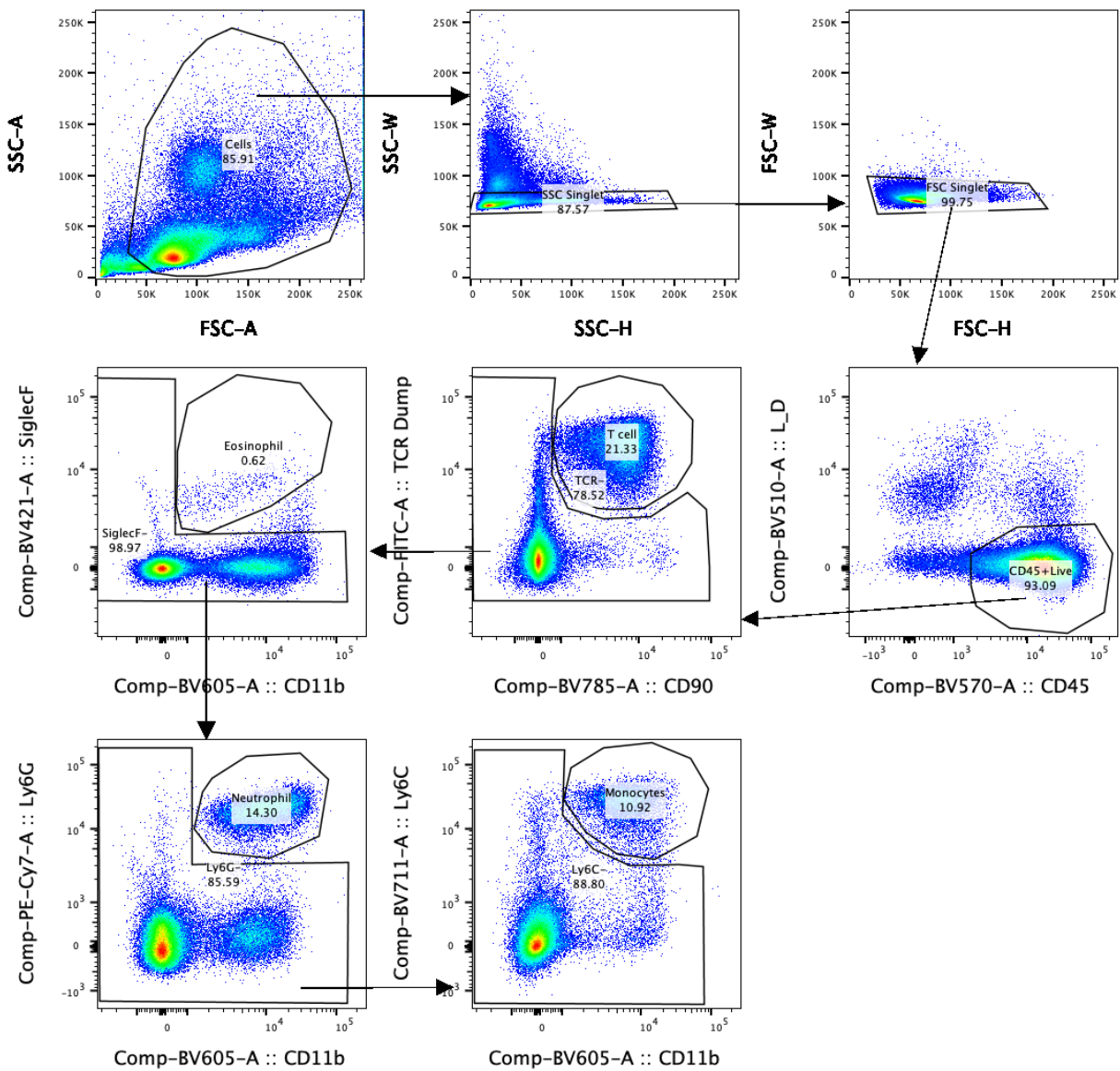

**Supplementary Figure 1 | Gating strategies for myeloid cell populations.**

Supplement: Supplementary Figure 1 [file NIHMS1820168-supplement-Supplementary_Figure_1.pdf]
